# A gut microbiota‐bile acid axis inhibits the infection of an emerging coronavirus by targeting its cellular receptor aminopeptidase N

**DOI:** 10.1002/imt2.70061

**Published:** 2025-07-03

**Authors:** Ya‐Qing Zhang, Bin Wang, Yong‐Le Yang, Jin‐Xin Meng, Meng‐Di Zhang, Yi‐Ke Li, Bo Dong, Yanan Zhang, Bo‐Wen Liu, Dong Yang, Chun‐Miao Ji, Yao‐Wei Huang, Shu Jeffrey Zhu

**Affiliations:** ^1^ State Key Laboratory for Animal Disease Control and Prevention South China Agricultural University Guangzhou China; ^2^ Department of Veterinary Medicine, College of Animal Sciences Zhejiang University Hangzhou China; ^3^ Xianghu Laboratory Hangzhou China; ^4^ Guangdong Laboratory for Lingnan Modern Agriculture, College of Veterinary Medicine South China Agricultural University Guangzhou China; ^5^ Agro‐biological Gene Research Center of Guangdong Academy of Agricultural Sciences State Key Laboratory of Swine and Poultry Breeding Industry Guangzhou China

**Keywords:** aminopeptidase N, *Bacteroides fragilis*, lithocholic acid, metabolomic, microbiota dysbiosis, porcine deltacoronavirus

## Abstract

Porcine deltacoronavirus (PDCoV) is a significant pathogen of swine with a global distribution, leading to severe gastrointestinal disease and substantial economic losses. Furthermore, PDCoV poses a potential threat to human health, as evidenced by the recent identification of three cases of infection in Haitian children. This study aimed to investigate the effects of PDCoV infection on host intestinal microbiota and bile acid metabolism, as well as the antiviral effects of lithocholic acid (LCA) in vitro and in vivo. Our results revealed that PDCoV infection caused microbiota dysbiosis in piglets, significantly reducing the intestinal abundance of *Bacteroides fragilis* (*B. fragilis*), a reduction that correlated with disruptions in bile acid metabolism. Colonization with bile salt hydrolase (BSH)‐producing *B. fragilis* increased the levels of unconjugated bile acids and inhibited PDCoV infection, highlighting the role of microbiota‐associated bile acid metabolism in viral pathogenesis. LCA, a prominent unconjugated bile acid, was shown to effectively inhibit PDCoV infection in porcine small intestinal epithelial cells and porcine intestinal enteroids. Notably, LCA inhibited PDCoV replication independently of bile acid receptor signaling and innate immune modulation. Mechanistic studies indicated that LCA prevents PDCoV infection by disrupting the viral entry process, specifically inhibiting the binding between the PDCoV spike protein and its cellular receptor, aminopeptidase N. In vivo experiments further confirmed that LCA significantly inhibited PDCoV infection in piglets. These results collectively highlight the potential of LCA as a therapeutic agent against PDCoV by targeting and disrupting the viral entry process, providing a novel strategy to control zoonotic PDCoV infections.

## Highlights

- Porcine deltacoronavirus (PDCoV) infection disrupts the gut microbiota homeostasis, which reduces *Bacteroides fragilis* (*B. fragilis*) abundance and impairs bile acid metabolism by suppressing the bile salt hydrolase activity, leading to decreased levels of unconjugated bile acids.
- Lithocholic acid (LCA) inhibits PDCoV by directly targeting the spike‐ aminopeptidase N binding interface, disrupting their interaction, independently of bile acid receptor signaling or innate immunity activation.
- In PDCoV‐infected piglets, oral LCA administration reduces viral shedding, alleviates clinical symptoms, and preserves intestinal barrier integrity, demonstrating its therapeutic potential in combating PDCoV infection.

## Graphical abstract

*Bacteroides fragilis* employs bile salt hydrolase to reprogram host bile acid metabolism, generating lithocholic acid that directly blocks porcine deltacoronavirus infection by disrupting spike protein‐aminopeptidase N binding, defining a microbiota‐bile acid antiviral axis with therapeutic potential against coronaviruses.

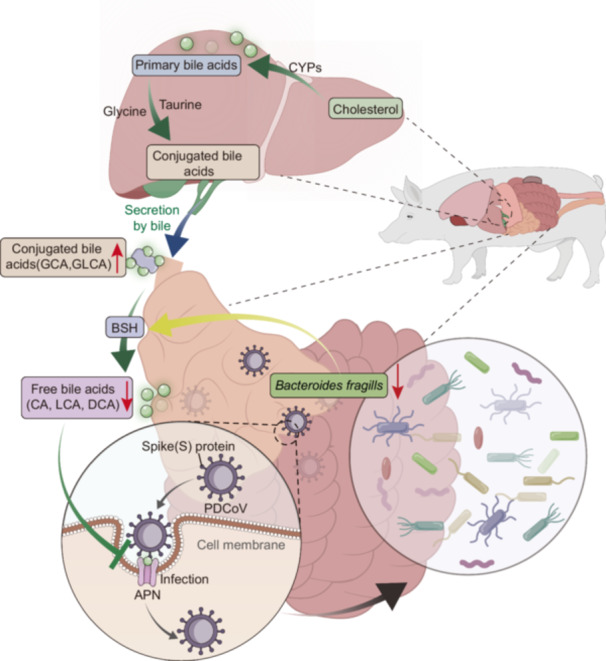

## INTRODUCTION

The gut microbiota, a diverse community of microorganisms inhabiting the gastrointestinal tract, regulates metabolic and immune homeostasis [1]. Additionally, it acts as a critical barrier against gastrointestinal pathogen invasion [2]. Gut microbiota‐derived metabolites, originating from bacterial synthesis, dietary sources, or microbial modification of host substrates [3], orchestrate intestinal homeostasis through cross‐species signaling [4], thereby shaping mucosal immunity and microbial colonization [5]. Among these metabolites, bile acids have emerged as key modulators of enteric viral infections. Primary bile acids, such as cholic acid (CA) and chenodeoxycholic acid (CDCA), are synthesized from cholesterol in the liver and released into the intestinal lumen after conjugation with taurine (TCA, TCDCA) or glycine (GCA, GCDCA). In the intestine, gut bacteria metabolize bile acids through a two‐step enzymatic process: First, bile salt hydrolases (BSH) deconjugate glyco‐ and tauro‐conjugated CA and CDCA, which are then 7α‐dehydroxylated to generate secondary bile acids—deoxycholic acid (DCA) and lithocholic acid (LCA). Bile acid‐activated receptors, such as the farnesoid X receptor (FXR) and the Takeda G protein‐coupled receptor 5 (TGR5), regulate bile acid homeostasis and modulate antiviral immune responses [6, 7]. Intriguingly, bile acids exhibit context‐dependent immunomodulatory effects, demonstrating both proviral and antiviral properties across different viral species. Mechanistically, bile acids facilitate viral entry of porcine enteric calicivirus, human noroviruses, and sapoviruses through host membrane remodeling and receptor clustering [8, 9, 10]. Conversely, bile acids can trigger antiviral responses, evidenced by their ability to reduce the infection of viruses such as murine cytomegalovirus (MCMV) and rotavirus, while increasing type I interferon (IFN) responses in certain viral infections [11, 12, 13, 14, 15]. Recent studies illuminate the role of bile acids in controlling enteric viral infections, highlighting their potential as therapeutic targets [16].

Emerging studies investigate how bile acids influence swine enteric coronaviruses (SECoVs) infections, including porcine epidemic diarrhea virus (PEDV), swine acute diarrhea syndrome coronavirus (SADS‐CoV), and porcine deltacoronavirus (PDCoV) [17]. For instance, using a novel porcine intestinal enteroid (PIE) model, we previously demonstrated that CA enhances early‐stage SADS‐CoV infection [18]. This virus was first identified in Guangdong, China, in 2017 [19]. However, recent findings indicate that certain strains of *Lactobacillus* isolated from local Chinese porcine species can convert primary bile acids into secondary bile acids, such as LCA, thereby triggering an immune response against PEDV [20]. These seemingly contradictory results suggest that different kinds of bile acids may modulate the infectivity of SECoVs through distinct mechanisms. Unfortunately, the underlying mechanisms by which bile acids regulate PDCoV infection in intestinal epithelial cells (IECs) and in vivo remain unclear. PDCoV, an enveloped single‐stranded positive‐sense RNA virus, is classified as a member of the genus *Deltacoronavirus* within the family *Coronaviridae* [21, 22]. First detected in surveillance samples from Hong Kong in 2012 [23], this pathogen was subsequently confirmed as the causative agent of diarrhea outbreaks affecting swine populations in the United States in 2014, with subsequent global reports documenting its widespread distribution [17, 24, 25]. PDCoV can cause severe watery diarrhea, vomiting, dehydration, and high mortality rates in newborn piglets worldwide, resulting in significant economic losses [24, 26, 27, 28]. Notably, experimental studies have demonstrated PDCoV's broad species tropism, with successful infections reported in calves, chickens, mice, and turkeys [29, 30, 31, 32]. Recently, PDCoV strains were first identified in plasma samples from three Haitian children with acute undifferentiated febrile illness [33], highlighting the potential risk for zoonotic transmission and public health concerns. This cross‐species transmission is mediated by conserved interactions between the PDCoV spike (S) protein and aminopeptidase N (APN) orthologues across species, including pigs, chickens, mice, and humans [31, 34, 35].

Here, we screened the gut microbiota of PDCoV‐infected pigs and identified that the abundance of the BSH‐carrying commensal bacterium *Bacteroides fragilis* (*B. fragilis*) was significantly reduced upon PDCoV infection. Using targeted metabolomics analysis of serum samples from PDCoV‐infected piglets, we revealed that PDCoV infection significantly reduced most bile acids, particularly unconjugated bile acids such as LCA and DCA. The observed reduction demonstrated a significant positive correlation with PDCoV‐induced declines in the relative abundance of *B. fragilis*. Utilizing a porcine intestinal epithelial cell line (IPEC‐J2) and a PIE infectious model in vitro, we demonstrate that LCA inhibits PDCoV infection primarily by interfering with the binding of the PDCoV spike protein to its cellular receptor, porcine APN (pAPN), rather than by activating antiviral innate immune responses. Our findings highlight the therapeutic potential of LCA as a dietary intervention against PDCoV infection, which warrants further investigation.

## RESULTS

### Alteration of gut microbiome landscape associated with PDCoV infection

**FIGURE 1:**
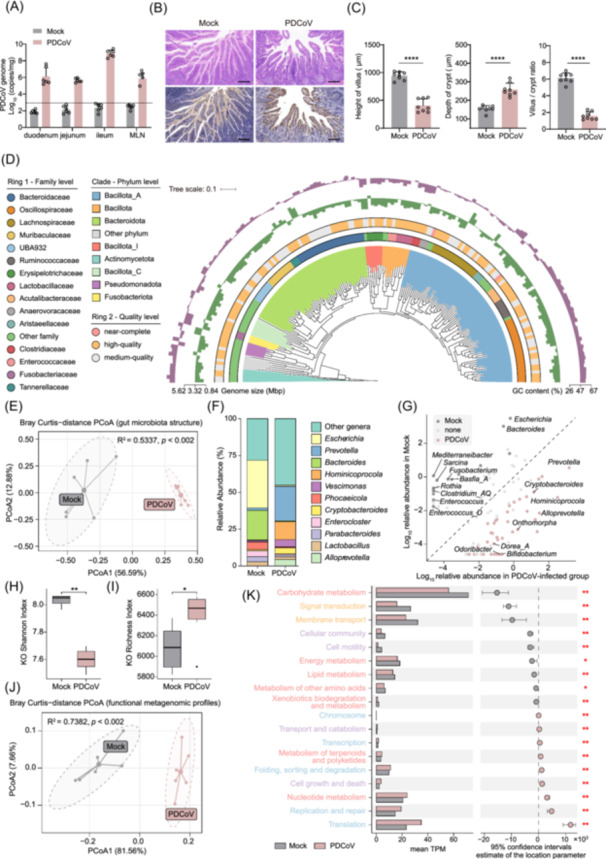
Effect of porcine deltacoronavirus (PDCoV) infection on the balance of intestinal microbiota. (A) Detection of viral load in the small intestine and mesenteric lymph nodes (MLN) at 3 days postinfection (dpi). (B) Histopathological changes (H&E staining) and PDCoV nucleocapsid (N) protein immunostaining in ileal tissues at 5 dpi. (magnification: ×100, scale bar: 100 μm). (C) Measurements of villus height and crypt depth. Two‐tailed Student's *t*‐test: ****p ≤ 0.0001. (D) Phylogenetic tree of 171 species‐level genomes. Clade colors correspond to phylum‐level classification. The first outer ring displays family‐level taxonomy, the second outer ring indicates genome quality scores, and the third and fourth outer rings represent genome size and GC content in bar plots, respectively. Medium‐quality: completeness >50% and contamination <10%; high‐quality: completeness >90% and contamination <5%; near‐complete: completeness >90%, contamination <5%, and presence of the 23S, 16S, and 5S rRNA genes and at least 18 tRNAs. (E) Principal coordinates analysis (PCoA) scatter plot of gut microbiota structure based on Bray–Curtis distances. Samples are plotted along the first two principal coordinates (PCoA1 and PCoA2), with variance explained by each axis indicated. Ellipses represent 95% group‐specific confidence intervals. PERMANOVA (999 permutations) tested the effect of PDCoV infection. (F) Genus‐level composition of the pig gut microbiota in PDCoV‐infected and control groups. (G) Scatter plot of log_10_‐transformed relative abundance of microbial genera in PDCoV‐challenged and healthy pigs. Boxplots of Shannon diversity (H) and feature richness (I) indices for Kyoto Encyclopedia of Genes and Genomes (KEGG) Orthologs (KOs) in PDCoV‐infected and the control piglets. Wilcoxon rank‐sum test: **p < 0.01, *p < 0.05. (J) PCoA scatter plot reflecting β‐diversity of functional metagenomic profiles in the pig gut microbiome. (K) Combined bar‐scatter plot showing abundance variations and statistically significant functional features between PDCoV‐infected and control groups. The tick labels on the *y*‐axis are color‐coded to represent different KEGG pathway categories: red for metabolism, orange for environmental information processing, purple for cellular processes, and blue for genetic information processing. Wilcoxon rank‐sum test with BH‐adjusted *p*‐value: **p < 0.01, *p < 0.05.

Twelve 3‐day‐old healthy piglets were randomly divided into two groups: the infected group received an oral inoculum of PDCoV (10^6^ TCID_50_/mL), and the control group was administered Dulbecco's Modified Eagle's Medium (DMEM). Infected piglets developed severe diarrhea at 2 days postinfection (dpi), whereas control piglets remained clinically healthy during the experimental period (Figure S1A). At 5 dpi, gross examination of PDCoV‐infected piglets revealed marked intestinal pathology, including distension of the small intestine with thin, transparent walls and accumulation of yellowish watery luminal contents (Figure S1B). Reverse transcriptase quantitative polymerase chain reaction (RT‐qPCR) analysis demonstrated significant viral replication in the small intestines and mesenteric lymph nodes (MLN), with the highest viral loads localized to the ileum (Figure 1A). Histopathological analysis via hematoxylin and eosin staining revealed severe architectural disruption in PDCoV‐infected intestines, characterized by significant villus shortening and crypt hyperplasia, consistent with loss of intestinal homeostasis. Ileal villi in infected piglets displayed atrophy and fragmentation, contrasting with intact mucosal architecture in controls (Figure 1B,C). Immunohistochemical (IHC) staining further confirmed PDCoV infection, showing strong positivity for the viral nucleocapsid (N) protein in ileal epithelial cells of infected piglets, whereas controls were negative (Figure 1B).

To assess the impact of PDCoV infection on gut microbiota composition, we performed 16S rRNA gene sequencing of fecal samples from PDCoV‐infected piglets and mock‐infected controls (treated with DMEM). Overall, within‐sample diversity indices revealed that PDCoV infection significantly increased the diversity in the pig gut community (effect size > 0.8, *p* < 0.01, Figure S1C). Beta diversity analysis using principal coordinates analysis (PCoA) based on Bray–Curtis distances demonstrated significant structural divergence between groups (PERMANOVA with 999 permutations, *R* ^*2*^ = 0.4846, *p* < 0.003, Figure S1D). Taxonomic profiling revealed a distinct clustering of gut microbiota between PDCoV‐infected and control groups at both phylum (Figure S1E) and genus (Figure S1F) levels. According to the results of linear discriminant analysis (LDA) effect size (LEfSe), the PDCoV‐infected group exhibited a significantly higher relative abundance of Firmicutes (*LDA* score = 5.29, Benjamini–Hochberg (BH)‐adjusted *p* < 0.05), and a significantly lower relative abundance of Proteobacteria (*LDA* score = 5.27, BH‐adjusted *p* < 0.05) and Fusobacteriota (*LDA* score = 4.79, BH‐adjusted *p* < 0.05) compared with the mock group (Figure S1G and Table S1). Genus‐level analysis further indicated that PDCoV infection significantly reduced the relative abundances of *Escherichia‐Shigella* and *Fusobacterium*, while promoting the enrichment of *Lactobacillus* and *Collinsella* (*LDA* score > 4, BH‐adjusted *p* < 0.05).

To overcome the low taxonomic resolution of 16S rRNA amplicon sequencing, we conducted shotgun metagenomic sequencing to deeply characterize the gut microbiome in PDCoV‐infected piglets. Using an assembly‐based approach, we reconstructed 479 medium‐ and high‐quality metagenome‐assembled genomes (MAGs) with an average completeness of 86.7% (±13.4%) and an average contamination of 1.4% (±1.8%, Figure 1D, Table S2). Taxonomic annotation revealed that these MAGs spanned 14 phyla, 44 families, 105 genera, and 171 genome‐based species (ANI > 95%). Subsequent profiling of the gut microbial community revealed remarkable variation in both diversity and richness (effect size > 0.78, *p* < 0.01), closely mirroring the trends observed in the 16S rRNA‐based characterization (Figure S2A). Specifically, Bray–Curtis distance‐based PCoA revealed that the gut microbiome structure was significantly altered following the PDCoV challenge (PERMANOVA with 999 permutations, *R* ^*2*^ = 0.5337, *p* < 0.002, Figure 1E). These changes were characterized by an increase in the relative abundance of Bacillota_I (effect size = 0.83, BH‐adjusted *p* < 0.01) and Bacillota_A (effect size = 0.60, *p* < 0.05), alongside a decrease in Pseudomonadota (effect size = 0.83, *p* < 0.01, formerly Proteobacteria) and Fusobacteriota (effect size = 0.60, *p* < 0.05) in PDCoV‐infected individuals (Figure S2B,C). At the genus level, we identified 11 health‐associated genera that were significantly depleted following infection (effect size > 0.71, BH‐adjusted *p* < 0.05, Figure 1F,G), including *Bacteroides*, *Escherichia*, and *Fusobacterium*. Conversely, 35 infection‐associated genera were significantly enriched in PDCoV‐infected piglets (effect size > 0.69, BH‐adjusted *p* < 0.05), such as *Hominicoprocola*, *Prevotella*, and *Alloprevotella*.

Using metagenome sequencing, we characterized the microbial functional landscape under PDCoV infection. Following the annotation of protein‐coding genes from 479 MAGs, 66.1% (272,184/411,474) of predicted genes were assigned to 7004 Kyoto Encyclopedia of Genes and Genomes (KEGG) Orthologs (KOs, Figure S2D). Within‐sample diversity analysis demonstrated that a significant reduction in functional heterogeneity (Shannon diversity) occurred in PDCoV‐infected piglets (effect size 0.84, *p* < 0.01), whereas KO richness (KO counts) showed an opposite trend (effect size = 0.79, *p* < 0.05, Figure 1H,I). Notably, Bray–Curtis distance‐based PCoA revealed that PDCoV infection exerted a more pronounced effect on microbial functional profiles than on taxonomic composition (PERMANOVA with 999 permutations, *R* ^*2*^ = 0.7382, *p* < 0.002, Figure 1J). Using the KO hierarchical classification, we quantified the relative abundance of functional categories and performed comparative analyses (Figure 1K and Figure S2E). Intriguingly, a majority of microorganism‐associated metabolic pathways showed significant depletion following PDCoV infection, specifically carbohydrate energy and lipid metabolism (effect size > 0.73, BH‐adjusted *p* < 0.05). In contrast, genetic information processing functions were markedly enhanced in infected piglets, particularly protein translation, folding, sorting, and degradation, as well as DNA replication and repair pathways (effect size > 0.83, BH‐adjusted *p* < 0.01). Altogether, these findings demonstrate that PDCoV infection induces substantial shifts in both gut microbial diversity and functional potential.

### Gut commensal *B. fragilis* was closely associated with PDCoV replication

**FIGURE 2:**
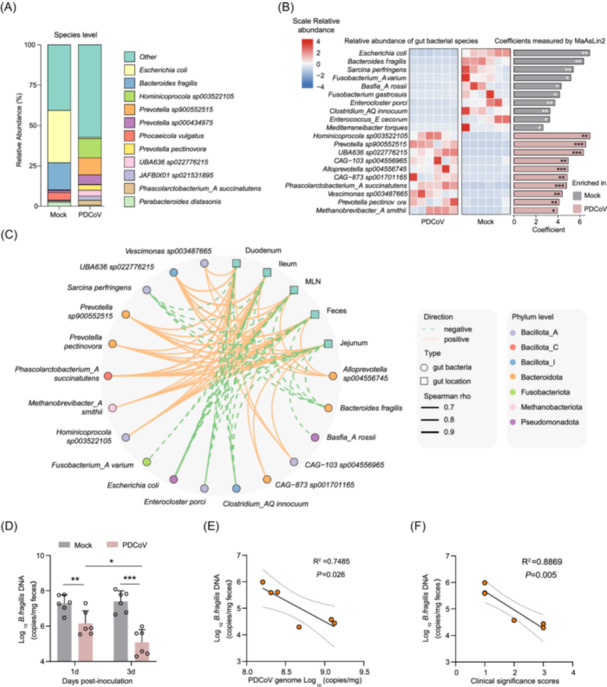
Porcine deltacoronavirus (PDCoV) replication is associated with depletion of Bacteroides fragilis (*B. fragilis*). (A) Species‐level composition of the pig gut microbiota in PDCoV‐infected and control groups. (B) Combined heatmap and bar plot showing the relative abundance of the top 20 differentially abundant species, alongside their Microbiome Multivariable Associations with Linear Models (MaAsLin2) model‐fitted values. MaAsLin2 with BH‐adjusted *p* values: ***p < 0.001, **p < 0.01, *p < 0.05, ^#^ *p* < 0.1. (C) Network diagram depicting correlations between the top 20 differentially abundant bacterial species and viral loads across intestinal segments. Orange solid lines represent significant positive correlations; green dashed lines signify significant negative correlations (BH‐adjusted *p* < 0.05). Circular nodes represent bacterial species colored by phylum. (D) Absolute abundance of *B. fragilis* at different time points in PDCoV‐infected piglets. Two‐tailed Student's *t*‐test: ***p < 0.001, **p < 0.01, *p < 0.05. Two‐tailed Pearson correlation analyses were performed between PDCoV copy numbers (E), clinicopathological scores (F), and *B. fragilis* abundance in the ileum.

We used high‐resolution metagenomics to characterize the piglet gut microbiota at the species level. We found that *Escherichia coli* (*E. coli*, 16.6%), *B. fragilis* (8.4%), *Hominicoprocola sp003522105* (6.1%), and 16 other species (relative abundance above 1.0%) dominated in the piglet gut microbiota (Figure 2A). Next, we identified significant shifts in microbial composition following PDCoV infection using Microbiome Multivariable Associations with Linear Models (MaAsLin2). Among all detected species, 55 (34.4%) showed significant enrichment in PDCoV‐infected piglets, while 15 (9.4%) were depleted (BH‐adjusted *p* < 0.05, Table S3). Notably, *E. coli* and *B. fragilis* demonstrated the most pronounced depletion (coefficient > 6, BH‐adjusted *p* < 0.001), whereas *Hominicoprocola sp*. and *Prevotella sp*. exhibited the most substantial enrichment (coefficient > 6, BH‐adjusted *p* < 0.001, Figure 2B).

To evaluate potential associations between microbial species and PDCoV replication, we conducted Spearman's rank correlation analysis comparing the relative abundance of the top 20 differentially abundant species with viral loads across multiple intestinal compartments, feces, and MLN. Our analysis revealed 77 significant correlations involving 17 microbial species (BH‐adjusted *p* < 0.05, Spearman *r*_s_ > 0.6, Figure 2C). Notably, *B. fragilis*, *Clostridium_A innocuum*, and *E. coli* showed strong negative correlations with PDCoV loads across all five sample types. Conversely, *Prevotella spp*., *Alloprevotella sp*., and *Hominicoprocola sp*. demonstrated significant positive correlations with PDCoV loads. These findings were partially supported by complementary 16S rRNA analyses at the genus level (Figure S1H).

*B. fragilis* is a keystone commensal bacterium critical for maintaining intestinal barrier integrity and immune modulation [36, 37]. Its depletion during PDCoV infection likely exacerbates gut dysbiosis and viral pathogenesis. To directly verify these observational correlations, we analyzed *B. fragilis* colonization dynamics with disease progression. Absolute abundance measurements confirmed progressive loss of *B. fragilis* in infected animals during infection (Figure 2D), while mock controls maintained stable bacterial levels, directly linking PDCoV infection to this microbial depletion. Critically, the magnitude of *B. fragilis* reduction aligned with clinical severity: its abundance showed strong inverse correlations with ileal viral loads (Pearson *R* ^*2*^ = 0.7485, *p* = 0.026, Figure 2E) and pathological scores (*R* ^*2*^ = 0.8869, *p* = 0.005, Figure 2F). The concurrent decline in *B. fragilis* abundance and progression of infection severity further supports its potential role in counteracting PDCoV pathogenesis.

### PDCoV‐induced *B. fragilis* depletion drives conjugated bile acid accumulation through impaired bacterial deconjugation

**FIGURE 3:**
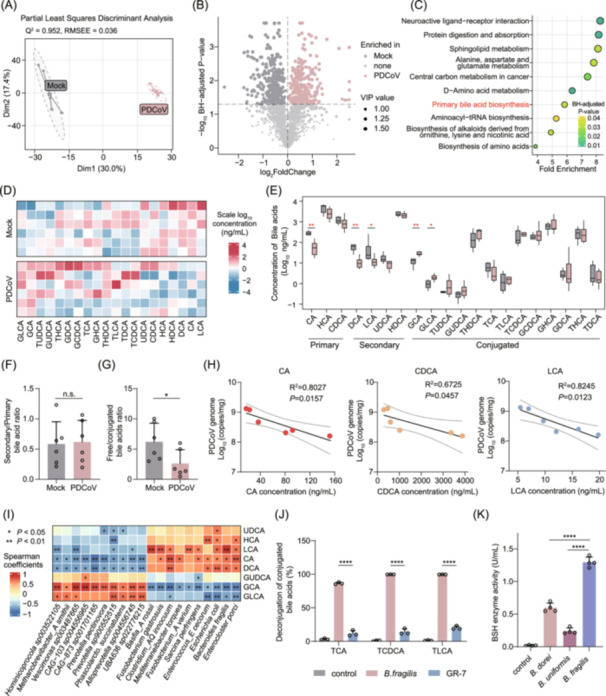
Significant changes in intestinal bile acids of piglets following oral porcine deltacoronavirus (PDCoV) infection. (A) Partial least squares discriminant analysis (PLS‐DA) of the piglet fecal metabolome. (B) Volcano plot of differentially abundant fecal metabolites (variable importance in projection (VIP) > 1 and BH‐adjusted *p* < 0.05). The size of each point represents the VIP value. The *y*‐axis scale corresponding to the horizontal line is −log_10_ (0.05). The statistical significance was determined using Welch's *t*‐test applied to log_10_‐transformed metabolite profiles, with Benjamini–Hochberg (BH) correction for multiple testing. (C) A scatter plot of KEGG pathway enrichment analysis for differentially abundant metabolites between PDCoV‐infected and healthy piglets. Statistical significance was determined using the hypergeometric test with BH correction for multiple testing. (D) Heatmap analysis of relative abundances of different bile acids. (E) Serum bile acid concentrations in PDCoV‐infected and mock‐infected piglets. Boxes represent the interquartile range between the first and third quartiles and the median (internal line). One‐way ANOVA: **p < 0.01, *p < 0.05. (F) The ratio of secondary bile acids/primary bile acids. (G) The ratio of free bile acids/conjugated bile acids. Two‐tailed student's *t*‐test: *p < 0.05, n.s., not significant. *p* > 0.05. (H) Pearson correlation between ileal PDCoV copy number and concentration of various bile acids. (I) Heatmap showing the correlation between PDCoV‐infected gut microbiota species and unconjugated bile acid levels. Spearman's rank test: **p < 0.01, *p < 0.05. (J) Hydrolytic efficiency of taurocholic acid (TCA), taurochenodeoxycholic acid (TCDCA), and Taurolithocholic acid (TLCA) by *B. fragilis* in the presence or absence of gut restricted‐7 (GR‐7) in vitro. Two‐tailed Student's *t*‐test: ****p ≤ 0.0001. (K) Concentrations of amino acids released from TCA, TCDCA, and TLCA, reflecting bile salt hydrolase (BSH) enzymatic activity. One‐way ANOVA with Dunnett's multiple comparisons test: ****p ≤ 0.0001.

Given the significant difference in microbial diversity and metagenomic function between PDCoV‐challenged and mock piglets, we further investigated microbial metabolite profiles using nontargeted metabolomics on collected fecal samples. Partial Least Squares Discriminant Analysis (PLS‐DA) revealed that PDCoV infection visibly impacted the microbial metabolic balance (*Q* ^2^ = 0.952, Square root of the mean error = 0.036, Figure 3A), supporting metagenome‐based findings. Subsequently, we identified 27.6% (560/2,030) of metabolites that showed significant variations following PDCoV infection (variable importance in projection (VIP) > 1 and BH‐adjusted *p* < 0.05), including 273 upregulated and 287 downregulated metabolites (Figure 3B). To determine which biological pathways were involved in regulating these metabolites, we conducted KEGG pathway enrichment analyses on these differentially expressed metabolites. Neuroactive ligand‐receptor interaction and protein digestion and absorption exhibited significant enrichment (BH‐adjusted *p* < 0.01). Additionally, sphingolipid metabolism and alanine, aspartate, and glutamate metabolism were also significantly enriched during viral infection, reflecting widespread impacts on host metabolic networks. Intriguingly, the primary bile acid pathway was also significantly enriched among the differential pathways (BH‐adjusted *p* < 0.05), suggesting that bile acid metabolism may play a vital role in PDCoV infection, potentially linked to host physiological adjustments in response to viral challenge (Figure 3C).

To confirm this speculation, we conducted a targeted metabolomic analysis of bile acids. The results demonstrated that PDCoV‐infected piglets exhibited a general decrease in free bile acid concentrations in the small intestine compared to uninfected controls, with significant reductions in CA, DCA, and LCA showing significantly lower levels (*p* < 0.05). In contrast, certain conjugated bile acids showed an increasing trend, particularly glycolcholic acid (GCA) and glycollithocholic acid (GLCA), which were significantly increased (*p* < 0.05, Figure 3D,E). Notably, while the ratio of secondary to primary bile acids showed no significant difference between groups (*p* > 0.05, Figure 3F), the ratio of unconjugated to conjugated bile acids was significantly lower in the infected group versus controls (*p* < 0.05, Figure 3G). Collectively, both metabolic profiling analyses demonstrated that PDCoV infection significantly altered bile acid profiles, characterized by downregulation of free bile acids and upregulation of conjugated bile acids.

Motivated by the imbalance of free and conjugated bile acids, we re‐analyzed metagenomic data and quantified the abundance of *BSH* and the 7α‐dehydroxylase gene (*baiCD*), two key enzyme‐coding genes in the bile acid metabolic pathway. The results showed that the *BSH* gene abundance was significantly higher in the control group than in the infected group (effect size = 0.832, *p* < 0.01, Figure S3A). Although the *baiCD* gene exhibited a trend of relatively higher abundance in the control group, this difference did not reach statistical significance (*p* > 0.05, Figure S3B). These findings further corroborate the metabolomic results, indirectly suggesting that PDCoV infection may inhibit microbial BSH activity, impair the dissociation of conjugated bile acids, and ultimately disrupt the dynamic balance of the host bile acid pool. To investigate the viral infection–bile acid relationship, we analyzed correlations between ileal viral copy numbers and free bile acid concentrations. We found the PDCoV copy numbers showed a significant negative correlation with CA, CDCA, and LCA (*p* < 0.05, Figure 3H), whereas no correlation was observed with hyocholic acid (HCA), DCA, hyodeoxycholic acid (HDCA), and ursodeoxycholic acid (UDCA) (Figure S3C).

To further investigate the regulatory role of the gut microbiota in the bile acid metabolic network, we analyzed associations between differentially abundant microbial species and bile acids using Spearman correlation analysis. The results showed that the concentrations of free bile acids (CA, LCA, and DCA) in the small intestine were significantly positively correlated with the abundances of *E. coli*, *Enterocloster porci*, and *B. fragilis* (*p* < 0.05, Figure 3I). By integrating these findings with the previously observed downregulation of *BSH* gene abundance in the infected group (Figure S3A) and the metabolomic evidence of conjugated bile acid accumulation (Figure 3D–G), we propose that PDCoV infection may inhibit the intestinal colonization of *B. fragilis* and other bacteria, as well as their BSH enzyme activity, thereby impairing the hydrolysis of conjugated bile acids and ultimately leading to decreased concentrations of free bile acids (CA, LCA, DCA).

BSH is one of the most extensively studied microbial enzymes involved in bile acid metabolism, converting host‐produced conjugated bile acids into microbially modified unconjugated bile acids [38]. Accumulating evidence has shown that *B. fragilis* is a primary source of microbiota‐derived BSH [39, 40]. Therefore, we investigated whether *B. fragilis* exhibits high BSH activity that could directly mediate the deconjugation of bile acids in vitro. Indeed, we observed that *B. fragilis* mediated the deconjugation of conjugated bile acids, and this process was attenuated by the BSH inhibitor gut restricted‐7 (GR‐7) (Figure 3J). Consistent with this finding, BSH enzyme activity was detected in three *Bacteroides* species, and the total BSH activity in *B. fragilis* was significantly higher than that in the other *Bacteroides* species (Figure 3K). In summary, these data suggest that an intestinal microenvironment with low *B. fragilis* abundance might facilitate PDCoV infection in vivo, as the impairment of BSH activity‐mediated bile acid deconjugation.

### Colonization with BSH‐producing *B. fragilis* increases the unconjugated bile acid pool and inhibits PDCoV infection in a mouse model

**FIGURE 4:**
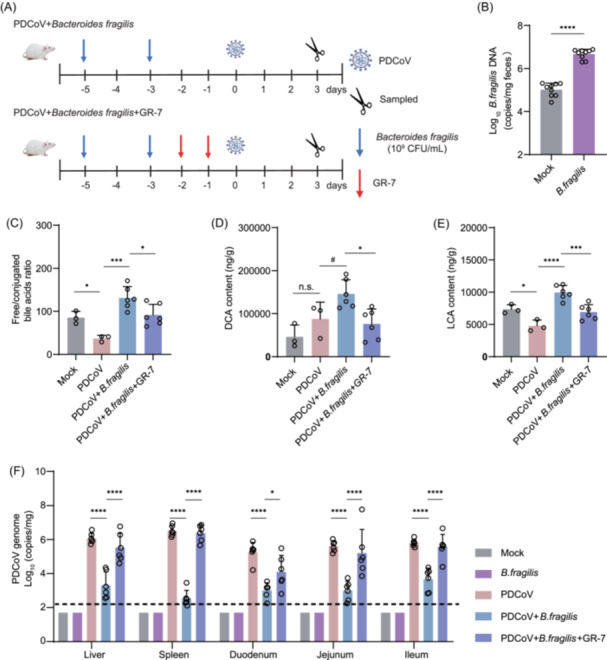
Impact of bile salt hydrolase (BSH)‐producing *B. fragilis* colonization on bile acid composition and porcine deltacoronavirus (PDCoV) dynamics. (A) Experimental design for in vivo colonization of *B. fragilis* and inhibition of BSH activity. (B) Fecal copy number of *B. fragilis* in mice 48 h after colonization. Two‐tailed Student's *t*‐test: ****p ≤ 0.0001. (C) The ratio of fecal unconjugated to conjugated bile acids in PDCoV‐infected mice at 3 dpi. One‐way ANOVA with Tukey's multiple comparisons test: ***p < 0.001, *p < 0.05. Fecal concentrations of the unconjugated bile acids deoxycholic acid (DCA) (D) and lithocholic acid (LCA) (E). One‐way ANOVA with Tukey's multiple comparisons test: ****p ≤ 0.0001, ***p < 0.001, *p < 0.05, ^#^ *p* < 0.1, n.s., not significant, *p* > 0.05. (F) Viral load was measured in the liver, spleen, duodenum, jejunum, and ileum of PDCoV‐infected mice at 3 dpi. Two‐way ANOVA with Tukey's multiple comparisons test: ****p ≤ 0.0001, *p < 0.05.

We next evaluated whether BSH‐producing *B. fragilis* restricted PDCoV infection in a mouse model. We colonized BALB/c mice with *B. fragilis* before PDCoV inoculation, since PDCoV can utilize murine APN as a receptor to infect mice [31]. To inhibit the BSH activity in vivo, we administered the BSH inhibitor GR‐7 to *B. fragilis*‐colonized mice (Figure 4A). Indeed, *B. fragilis* efficiently colonized the animals within 48 h post‐oral gavage (Figure 4B). Consistent with the observations in infected piglets, the ratio of unconjugated to conjugated bile acids in the feces of PDCoV‐infected mice decreased significantly. This decrease was reversed by *B. fragilis* colonization, but the reversal effect was significantly attenuated by GR‐7 (Figure 4C). Correspondingly, *B. fragilis* restored the fecal concentrations of unconjugated bile acids DCA and LCA, while GR‐7 diminished these concentrations in the presence of PDCoV infection (Figure 4D,E). The observed reduction in LCA levels following GR‐7 treatment may be attributed to the inhibition of BSH activity in multiple bacterial species, including but not limited to *B. fragilis*. Remarkably, colonization of BALB/c mice with *B. fragilis* reduced PDCoV infection in the liver, spleen, duodenum, jejunum, and ileum. In contrast, inhibition of BSH activity by GR‐7 nullified the antiviral effect of *B. fragilis* (Figure 4F). These findings further demonstrate the critical role of *B. fragilis*‐derived BSH activity in antagonizing PDCoV infection.

### LCA effectively inhibits PDCoV infection in porcine small intestinal epithelial cells (IPEC‐J2) and PIEs

**FIGURE 5:**
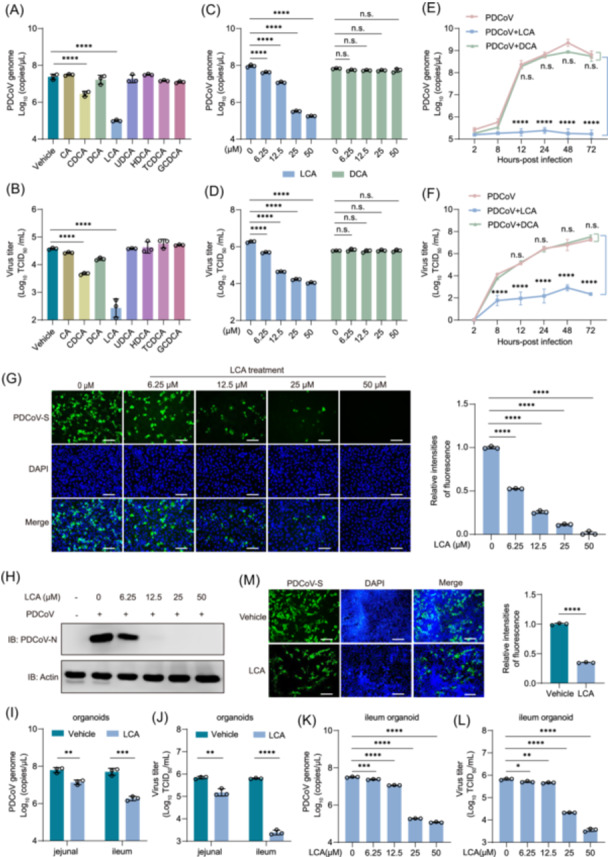
Lithocholic acid (LCA) inhibits porcine deltacoronavirus (PDCoV) infection in porcine small intestinal epithelial cells (IPEC‐J2) and porcine intestinal enteroids. (A) Viral RNA in PDCoV‐infected IPEC‐J2 cells was detected using reverse transcriptase quantitative polymerase chain reaction (RT‐qPCR). One‐way ANOVA with Dunnett's multiple comparisons test: ****p ≤ 0.0001. (B). Viral titers were determined using the standard TCID_50_ assay. One‐way ANOVA with Dunnett's multiple comparisons test: ****p ≤ 0.0001. (C, D) IPEC‐J2 cells were infected with PDCoV for 24 h in the presence of various concentrations of LCA and DCA. PDCoV viral loads and titers were determined using RT‐qPCR (C) and the TCID_50_ assay (D). Two‐way ANOVA with Dunnett's multiple comparisons test: ****p ≤ 0.0001, n.s., not significant, *p* > 0.05. (E, F) The infection dynamics of PDCoV‐infected IPEC‐J2 cells influenced by LCA or DCA were investigated using RT‐qPCR (E) and the TCID_50_ method (F). Two‐way ANOVA with Dunnett's multiple comparisons test: ****p ≤ 0.0001, n.s., not significant, *p* > 0.05. (G) IPEC‐J2 cells were infected with PDCoV (MOI = 0.1) and treated with different concentrations of LCA for 24 h. Cells were then stained with a mouse anti‐S protein antibody, followed by Alexa Fluor 488 anti‐mouse IgG and DAPI for nuclei. Images were captured using a fluorescence microscope (scale bar: 100 μm). Relative fluorescence intensity was quantified using Image software as shown on the right. One‐way ANOVA with Dunnett's multiple comparisons test: ****p ≤ 0.0001. (H) The expression level of the PDCoV N protein was assessed using western blot analysis. (I, J) Jejunal and ileal organoids were infected with PDCoV at an MOI of 0.1 in the presence and absence of LCA for 24 h. The viral load was assessed using RT‐qPCR (I), and infectious viral titer was determined using the TCID_50_ assay (J). Two‐tailed Student's *t*‐test: ****p ≤ 0.0001, ***p < 0.001, **p < 0.01. (K, L) Ileal organoids were infected with PDCoV at an MOI of 0.1 for 24 h in the presence of various concentrations of LCA. PDCoV viral loads and titers were determined using RT‐qPCR (K) and the TCID_50_ assay (L). One‐way ANOVA with Dunnett's multiple comparisons test: ****p ≤ 0.0001, ***p < 0.001, **p < 0.01, *p < 0.05. (M) The infected ileal organoids were fixed and subsequently subjected to IFA staining for the PDCoV S protein; nuclei were stained with DAPI (scale bar: 100 μm). The relative fluorescence intensity was quantified using Image software, as shown on the right. Two‐tailed Student's *t*‐test ****p ≤ 0.0001. DCA, deoxycholic acid; MOI, multiplicity of infection.

Although we observed a negative correlation between bile acid concentration and PDCoV copy number in vivo, whether bile acids regulate PDCoV infection remained unclear. To test this hypothesis, we first evaluated the cytotoxicity of various bile acids (primary, secondary, and conjugated) in IPEC‐J2 cells (Figure S4A) before investigating their role in PDCoV infection. After determining an optimal non‐cytotoxic concentration, we pretreated IPEC‐J2 cells with these bile acids before PDCoV infection. Following 24 h of incubation, RT‐qPCR and TCID_50_ assays were used to measure viral load and titer. The results showed that bile acids such as CDCA and LCA significantly inhibited PDCoV infection, with LCA exhibiting the most pronounced effect (Figure 5A,B). We then exposed the cells to different concentrations of LCA and observed a dose‐dependent inhibition of viral infection (Figure 5C,D). This finding was further supported by immunofluorescence assays (IFA) visualizing the PDCoV S protein and western blot analysis quantifying the N protein, both of which confirmed the dose‐dependent antiviral effect of LCA (Figure 5G,H). We also validated the effect of LCA on PDCoV infection in the porcine kidney cell line (LLC‐PK1), which is commonly used for PDCoV isolation and propagation. IFA and western blot indicated that PDCoV N and S protein levels significantly decreased in a dose‐dependent manner following LCA treatment (Figure S5A,B). To further validate LCA's inhibitory effect on PDCoV infection, we assessed the growth kinetics of LCA‐ and DCA‐treated PDCoV‐infected cells, along with untreated controls, at a multiplicity of infection (MOI) of 0.1. As shown in Figure 5E,F, from 8 to 72 h postinfection (hpi), the LCA‐treated group exhibited a significant decrease in viral copy number and titer, whereas the DCA‐treated group showed no significant difference compared to the control group. These findings indicate that LCA's inhibitory effect on viral infection occurs during the early stages.

To better model in vivo infection conditions, we used PIEs, which retain the structural and functional characteristics of natural intestinal epithelium, to conduct infection inhibition experiments. The results showed that LCA effectively inhibited PDCoV infection in both jejunal and ileal organoids (Figure 5I,J). In ileal organoids, LCA exhibited a dose‐dependent reduction in viral load and PDCoV titer compared to the control group (Figure 5K,L). Consistent with the viral load findings, the level of viral S protein in PDCoV‐infected ileal organoids was significantly lower in the LCA‐treated group than in the control group (Figure 5M).

### LCA inhibits PDCoV infection independently of bile acid receptor signaling and innate immune modulation

Previous studies have shown that bile acids enhance the antiviral immune response by interacting with membrane‐bound TGR5 and nuclear FXR receptors, thereby activating innate immune signaling pathways. To determine whether LCA exerts its antiviral effects through BAR signaling, we systematically evaluated the roles of FXR and TGR5 using pharmacological agonists, antagonists, and siRNA‐mediated knockdown. To establish safe and effective concentrations of FXR/TGR5 agonists and antagonists, we systematically assessed their cytotoxic effects on IPEC‐J2 cells using the CCK‐8 assay (Figure S4B,C). Treatment with LCA, FXR‐specific (INT‐747, 50 μM), TGR5‐specific (INT‐777, 50 μM), and dual FXR/TGR5 (INT‐767, 50 μM) agonists significantly upregulated mRNA levels of *FXR*, its downstream target small heterodimer partner (*SHP*), and *TGR5* (Figure S6A–C). LCA and TGR5 agonists (INT‐777, INT‐767) also elevated intracellular cAMP levels, confirming TGR5 pathway activation (Figure S6D). However, despite robust activation of BAR signaling, none of these agonists reduced PDCoV genome copies (Figure S6E), indicating that BAR signaling alone is insufficient to mediate antiviral activity. To further dissect LCA's mechanism, we employed antagonists: guggulsterone (GUG) to inhibit FXR and SBI‐115 to block TGR5. Pretreatment with these antagonists significantly suppressed LCA‐induced upregulation of *FXR*, *SHP*, and *TGR5* (Figure S6A–C), demonstrating effective yet partial attenuation of BAR signaling. Notably, neither antagonist impaired LCA's ability to inhibit PDCoV replication (Figure S6F,G), even at concentrations that substantially reduced BAR pathway activity. To exclude compensatory cross‐talk between *FXR* and *TGR5*, siRNA‐mediated knockdown of individual receptors (Figure S6H,I) disrupted their respective signaling pathways without diminishing LCA's antiviral efficacy (Figure S6J). Combined knockdown of both receptors also failed to counteract PDCoV inhibition (Figure S6J), further supporting a BAR‐independent mechanism.

Subsequently, we evaluated the modulation of the innate immune response. Poly (I:C) potently induced the expression of *IFNA*, *IFNB*, *IFNG*, and *IFNL1* at 24 hpi (Figure S7A–D). Conversely, LCA alone did not activate the IFN signaling pathways. Notably, PDCoV infection moderately upregulated *IFNB* and *IFNL1* expression; however, cotreatment with LCA abrogated PDCoV‐induced IFN production (Figure S6B,D). Similarly, while Poly (I:C) treatment and PDCoV infection significantly elevated the expression of interferon‐stimulated genes (*ISG15*, *IFITM1*, *MX1*, and *OAS1*), LCA alone or in combination with PDCoV did not alter the expression of these genes (Figure S7E–H). To conclusively rule out the involvement of IFN receptors, siRNA‐mediated knockdown of IFNAR1 or IFNLR1 (Figure S7I,J) did not attenuate LCA's inhibitory effect on PDCoV replication (Figure S7K). Taken together, these findings elucidate that LCA inhibits PDCoV infection independent of canonical FXR/TGR5 signaling pathway activation and IFN‐dependent innate immune mechanisms, highlighting its distinct antiviral mechanism of action.

### LCA prevents PDCoV infection by disrupting the viral entry process

**FIGURE 6:**
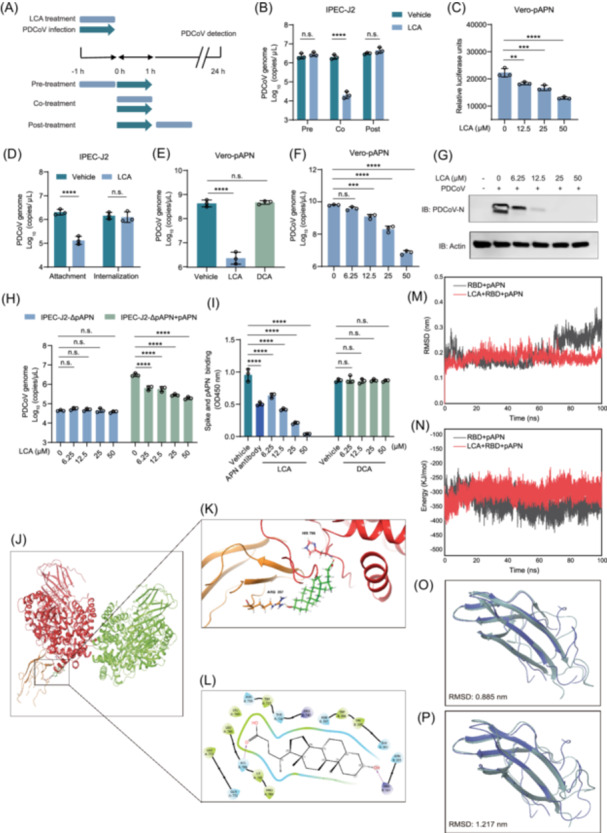
Lithocholic acid (LCA) inhibits porcine deltacoronavirus (PDCoV) infection by disrupting viral entry by blocking spike‐pAPN interaction and inducing conformational changes in the receptor‐binding domain (RBD)‐pAPN complex. (A) The schematic illustrates the timing of LCA addition in PDCoV infection. (B) LCA treatment timing in PDCoV‐infected IPEC‐J2 cells (MOI = 0.1). Viral RNA loads were quantified by reverse transcriptase quantitative polymerase chain reaction (RT‐qPCR) at 24 hpi. Two‐tailed Student's *t*‐test: ****p ≤ 0.0001, not significant, *p* > 0.05. (C) LCA‐mediated inhibition of PDCoV entry was evaluated using HIV pseudoviruses displaying PDCoV spike protein (HIV‐PDCoV‐S). Spike‐dependent cellular entry efficiency was quantified by luciferase activity at 48 hpi. One‐way ANOVA with Dunnett's multiple comparisons test: ****p ≤ 0.0001, ***p < 0.001, **p < 0.01. (D) Effect of LCA on the processes of viral attachment to or internalization into IPEC‐J2 cells. Two‐tailed Student's *t*‐test: ****p ≤ 0.0001, not significant, *p* > 0.05. (E, F) Detection of PDCoV viral load in Vero cells overexpressing the cellular receptor porcine APN (Vero‐pAPN) using RT‐qPCR. (E) Vero‐pAPN cells were infected with PDCoV (MOI = 0.1) for 24 h in the presence of LCA and DCA. (F) Vero‐pAPN cells were infected with PDCoV (MOI = 0.1) for 24 h in the presence of different concentrations of LCA. One‐way ANOVA with Dunnett's multiple comparisons test: ****p ≤ 0.0001, ***p < 0.001, n.s., not significant, *p* > 0.05. (G) The expression level of the PDCoV N protein was assessed using western blot analysis. (H) The effect of LCA on PDCoV‐infected pAPN‐deficient IPEC‐J2 cells (IPEC‐J2‐ΔpAPN) and pAPN‐supplemented IPEC‐J2‐ΔpAPN cells was assessed using RT‐qPCR. Two‐way ANOVA with Dunnett's multiple comparisons test: ****p ≤ 0.0001, n.s., not significant, *p* > 0.05. (I) The interaction between the PDCoV spike protein and pAPN was assessed using an enzyme‐linked immunosorbent assay (ELISA). Two‐way ANOVA with Dunnett's multiple comparisons test: ****p ≤ 0.0001, n.s., not significant, *p* > 0.05. Global interaction diagram (J), local interaction diagram (K), and 2D diagram (L) of the LCA‐RBD‐pAPN protein complex after docking. (M) The curve shows the change in protein RMSD over time during the protein‐ligand complexation simulation. (N) The interaction energy curve between the protein and ligand was plotted during the simulation. (O, P) Simulation of protein and ligand complex formation. (O) The simulated complex of RBD and pAPN proteins alone, and (P) simulated complex of RBD and pAPN proteins in the presence of LCA (purple denotes the initial protein model at the start of the simulation, while turquoise represents the protein model after 100 ns). MOI, multiplicity of infection.

To determine the stage at which LCA inhibits PDCoV infection in IPEC‐J2 cells, we treated the cells with LCA 1 h before (pretreatment), during (co‐treatment), or 1 h after (post‐treatment) PDCoV inoculation (Figure 6A). Following LCA treatment and virus incubation, the cells were transferred to fresh medium for further incubation. At 24 hpi, cells were harvested for RNA extraction and viral load was determined by RT‐qPCR. We found that neither pretreatment nor posttreatment with LCA significantly affected PDCoV load. Notably, co‐treatment with LCA and PDCoV resulted in a significant reduction in viral load at 24 hpi, suggesting that LCA exerts its antiviral effect during the viral entry stage (Figure 6B). Furthermore, we demonstrated that LCA effectively inhibits the entry of PDCoV spike protein pseudovirus (HIV‐PDCoV‐S), a pseudotyped retrovirus displaying the PDCoV spike on the surface, into Vero cells overexpressing APN (Vero‐pAPN). This finding provides further evidence that LCA directly blocks cell entry mediated by the PDCoV spike protein (Figure 6C).

We next examined the effect of LCA on PDCoV attachment and internalization. For the attachment assay, IPEC‐J2 cells were incubated with PDCoV at 4°C for 1 h in the presence or absence of LCA. This low‐temperature condition permitted viral binding to the cell surface while preventing internalization. Unbound virions were removed by washing with PBS, and the cells were maintained in culture medium for 24 h. For the internalization assay, cells were first incubated with PDCoV at 4°C for 1 h to allow viral attachment. Following the removal of unbound virus with cold PBS, the cells were warmed to 37°C and maintained in LCA‐containing medium for 1 h to permit internalization. The results indicated that LCA treatment significantly reduced virus attachment compared with the control group, demonstrating that LCA effectively inhibits PDCoV attachment to host cells (Figure 6D). However, in the internalization assay, LCA treatment after attachment did not alter the PDCoV load at 24 hpi (Figure 6D). These findings suggest that LCA may interfere with the binding of the PDCoV spike to host receptor proteins. The same experiment was conducted in porcine ileal organoids, and the results were consistent with those obtained in IPEC‐J2 cells (Figure S8A,B).

### LCA inhibits the binding of PDCoV spike proteins to pAPN

After confirming that LCA interacts with the virus during the attachment stage, we hypothesized that it might inhibit the interaction between spike proteins and pAPN. To explore this possibility, we conducted experiments in Vero cells overexpressing pAPN. The results were consistent with those observed in IPEC‐J2 cells, demonstrating that LCA significantly inhibited PDCoV infection in Vero‐pAPN cells, while DCA had no significant effect (Figure 6E). We treated Vero‐pAPN cells with various concentrations of LCA and used RT‐qPCR, IFA, and western blot analysis to measure viral replication, N protein expression, and S protein expression (Figure 6F,G, and Figure S8C). Collectively, these results indicated that LCA effectively inhibited PDCoV infection in a dose‐dependent manner in Vero‐pAPN cells.

In subsequent studies, we utilized an IPEC‐J2 cell line with pAPN knocked out (IPEC‐J2‐ΔpAPN) to investigate the effect of LCA on PDCoV infection. Surprisingly, we found that LCA's inhibitory effect on PDCoV infection was abolished in these cells, suggesting that pAPN is crucial for LCA inhibition of PDCoV infection. To confirm this, we reintroduced the pAPN into IPEC‐J2‐ΔpAPN cells and observed that LCA regained its ability to inhibit PDCoV infection (Figure 6H). Additionally, using an enzyme‐linked immunosorbent assay (ELISA), we confirmed that LCA inhibits the binding of the PDCoV S protein to pAPN (Figure 6I), whereas DCA did not affect this binding.

To further dissect the molecular mechanism, we conducted molecular docking studies of LCA with the receptor‐binding domain (RBD)‐pAPN protein complex. LCA exhibited a high affinity for the proteins, with a binding energy of −5.165 Kcal/mol (Figure 6J). The hydroxyl group of LCA interacts with N355, R357, T393, V395, W396, and N397 in the RBD, while the carboxyl group binds to P784, I785, H786, L789, N736, W737, T738, and R740 in the pAPN. LCA forms hydrogen bonds with R357 in the RBD protein and H786 in the pAPN protein, while the remaining amino acids interact via van der Waals forces with LCA. (Figure 6K,L). R357, T393, V395, W396, N397, and P784, I785, H786 are the key amino acid sites involved in RBD‐pAPN binding, and DCA cannot bind to this pocket (Figure S9A), which may explain why DCA does not affect PDCoV infection in cells. To determine whether LCA binding to RBD blocks its interaction with pAPN, we performed kinetic simulations and used root mean square deviation (RMSD) to measure coordinate deviation. A stable RMSD indicates that the atoms have reached equilibrium and are fluctuating within an acceptable range. As shown in Figure 6M, both the protein system and the protein‐ligand system reached equilibrium after a certain period, confirming the reliability of the entire simulation.

To investigate the interaction between the viral S protein and LCA, we first analyzed the interaction energy of the RBD‐pAPN complex in the presence or absence of LCA. In the absence of LCA, the interaction energy of the RBD‐pAPN complex was −338.03 KJ/mol, which decreased to −302.46 kJ/mol upon LCA addition (Figure 6N). This reduction in interaction energy indicates that LCA influences the interaction between the RBD and pAPN. To examine the impact of LCA on RBD structure, we analyzed conformational changes of RBD in the presence or absence of LCA using molecular dynamics (MDs) simulations. After 100 ns of simulation, the RMSD of RBD in the absence of LCA was 0.885 nm, increasing to 1.217 nm upon LCA addition (Figure 6O,P). The increase in RBD RMSD upon LCA addition suggests that LCA alters the protein's conformation. To validate these findings, we generated two S protein mutations, R357A and N355A, each harboring a single amino acid substitution. Competitive ELISA assays showed that LCA did not disrupt the interaction between the R357A mutant and pAPN (Figure S10A), but effectively blocked the binding of the N355A mutant protein (Figure S10B). We repeated molecular docking simulations with the R357A mutant and found that the LCA‐protein complex could not form hydrogen bonds with residue 357, and the binding energy decreased to −4.759 Kcal/mol (Figure S9B).

### LCA inhibits PDCoV binding to human aminopeptidase N (hAPN)

To assess the relevance of LCA's antiviral mechanism to humans, we investigated whether LCA disrupts PDCoV binding to hAPN. First, we transfected BHK‐21 cells with PRK5‐hAPN‐Flag (BHK‐hAPN) and confirmed that LCA significantly inhibited PDCoV replication in these cells, as evidenced by reduced viral RNA levels (Figure S11A) and decreased PDCoV N protein expression by IFA (Figure S11B). Molecular docking studies were performed to assess the interaction between LCA and the PDCoV RBD‐hAPN complex. LCA exhibited a binding energy of −4.837 Kcal/mol, forming hydrogen bonds with N397 in the viral RBD and N739 in hAPN (Figure S11C–E). This interaction mirrors the binding mode observed in pAPN, suggesting a conserved mechanism across species. These findings indicate that LCA's inhibitory effect on PDCoV entry is preserved in the context of hAPN, highlighting its potential relevance to zoonotic infections.

### LCA effectively inhibits PDCoV infection in piglets

**FIGURE 7:**
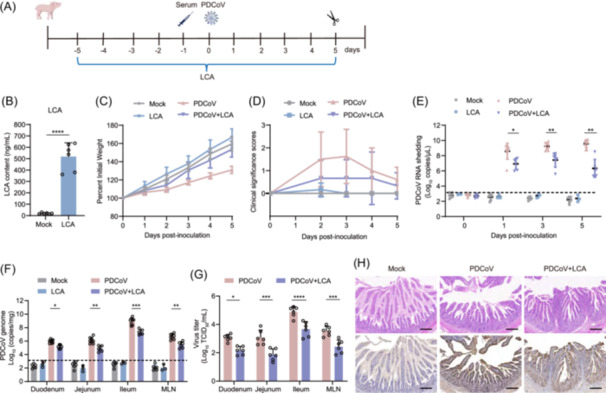
The effect of lithocholic acid (LCA) on porcine deltacoronavirus (PDCoV) infection in piglets. (A) Schematic diagram of the PDCoV infection experiment in piglets. (B) LCA levels in the serum of piglets after 5 days of oral administration. Two‐tailed Student's *t*‐test: ****p ≤ 0.0001. (C) Variations in body weight were observed in piglets over a specific period. (D) The scoring system for piglet diarrhea is as follows: 0 = normal; 1 = pasty stool; 2 = semi‐liquid diarrhea without viscosity (cow dung‐like feces); 3 = watery or liquid content (severe diarrhea). (E) RT‐qPCR detection of PDCoV fecal shedding in piglets. (F) The viral copy number of PDCoV in the small intestine and MLN was measured at 5 dpi. (G) Detection of infectious virus titer in the small intestine and MLN was performed using the TCID_50_ assay. (H) Histopathology and immunohistochemistry (IHC) analyses of the ileum in infected piglets at 5 dpi (magnification: ×100, scale bar: 100 μm). (E–G) *p*‐Values were determined using a mixed‐effects model: ****p ≤ 0.0001, ***p < 0.001, **p < 0.01, *p < 0.05. RT‐qPCR, reverse transcriptase quantitative polymerase chain reaction.

To verify LCA's protective effect in piglets, we orally administered LCA (30 mg/kg/day) to newborn piglets for five consecutive days, followed by inoculation with PDCoV (2 × 10^6^ TCID_50_) or DMEM on Day 0 (Figure 7A). As shown in Figure 7B, oral administration of LCA before PDCoV infection significantly increased serum LCA levels. To evaluate the safety of LCA as a prophylactic intervention, we examined intestinal barrier integrity, inflammatory responses, and apoptotic signaling in PDCoV‐infected and LCA‐treated piglets. Gene expression analysis of the ileum revealed no significant differences in intestinal barrier‐associated genes (*MUC2*, *OCCLUDIN*, *ZO1*, and *CLAUDIN1*), pro‐inflammatory cytokines (*IL6*, *IL8*, *IL1B*, and *TNFA*), or apoptosis‐related markers (*BAX*, *BCL2*, *CASP3*, and *CASP9)* between LCA‐treated and untreated groups (Figure S12A–H). These findings collectively indicate that LCA administration does not compromise intestinal barrier function or induce significant inflammatory or apoptotic responses in piglets under our experimental conditions. Additionally, piglets receiving LCA treatment following PDCoV infection exhibited improved clinical outcomes, including slightly faster weight gain (Figure 7C) and reduced diarrhea severity (Figure 7D) compared to infected, untreated controls. Notably, the PDCoV + LCA group exhibited substantially reduced viral shedding in feces, with statistically significant differences observed at 1, 3, and 5 dpi compared to the PDCoV‐only group (Figure 7E). Moreover, viral loads in the duodenum, jejunum, ileum, and MLN at 5 dpi were significantly reduced in the PDCoV + LCA group than in the untreated PDCoV‐infected group. Notably, infectious viral titers across all tissues were markedly lower in the LCA‐treated group. The ileum exhibited the highest viral replication efficiency, consistent with its role as the primary site of PDCoV infection due to high receptor (pAPN) expression and bile acid‐microbiota interactions (Figure 7F,G). Furthermore, IHC analysis of ileal tissues at 5 dpi revealed extensive PDCoV N protein expression in IECs of untreated infected animals, whereas the PDCoV + LCA group showed markedly reduced immunostaining (Figure 7H). To investigate the host antiviral response, we quantified IFN and IFN‐stimulated gene (ISG) expression in the ileum. Consistent with viral replication patterns, untreated PDCoV‐infected piglets demonstrated significant upregulation of *IFNB*, *IFNG*, and *IFNL1* interferons, accompanied by robust induction of ISGs (*MX1*, *ISG15*, *IFITM1*, and *OAS1*) (Figure S13A–H). In contrast, LCA treatment substantially attenuated this IFN/ISG response, with only minimal gene activation observed compared to infected controls.

## DISCUSSION

Bile acids are pivotal in lipid digestion, absorption, and modulating signaling pathways that impact gut microbiota and host metabolism [8, 41, 42]. As active mediators of innate immunity, they exhibit antiviral activities through interactions with specific BARs, such as FXR and TGR5 [6, 7]. For instance, bile acids and FXR agonists have been shown to reduce rotavirus infection in vitro and viral shedding in vivo [12]. Grau et al. demonstrated that bile acids inhibit murine norovirus infection in the proximal gut by promoting type III IFN expression, although this effect was not observed in the distal gut, likely due to regional variations in BAR expression [43]. These findings underscore the complex role of bile acids in antiviral defense, which is contingent upon receptor distribution and anatomical location.

Recent research has increasingly emphasized the intricate interplay between the gut microbiota, bile acids, and viral infections. Viral invasion can profoundly disrupt the gut microbial composition, as evidenced by PDCoV‐infected piglets, which show increased fecal *Lactobacillus* abundance and decreased *Bacteroides* levels [44]. Our 16S rRNA sequencing data align with this trend, revealing a genus‐level increase in *Lactobacillus* and decreases in *Bacteroides* and *Fusobacterium* in piglets infected with the PDCoV HZYH‐2019 strain compared to healthy controls (Figure S1F,G). However, these results contrast with Li et al.'s report, which showed reduced proportions of Firmicutes and Bacteroidetes in the jejunum of PDCoV‐infected piglets, along with increased *Fusobacterium* and decreased *Lactobacillus* at the genus level [45]. These discrepancies may be attributed to the distinct tropism of viral strains: CZ2020 preferentially colonizes the proximal jejunum, whereas HZYH‐2019 targets the distal ileum. Regional variations in microbial composition and metabolic activity, influenced by localized bile acid gradients or mucin secretion, could drive these strain‐specific microbiota shifts. Additionally, jejunal mucosal microbiota, as analyzed by Li et al., may be more vulnerable to acute immune perturbations, while fecal samples, used in our study, reflect colonic microbial communities where such effects may be mitigated.

Metagenomic profiling further elucidated the significant restructuring of gut microbial communities induced by PDCoV infection at both taxonomic and functional levels. Our analysis revealed a marked expansion of Firmicutes accompanied by suppression of *Pseudomonadota* and *Fusobacteriota*. These shifts were evident at the species level, including the enrichment of *Hominicoprocola sp*. and depletion of *E. coli* and *B. fragilis*, as well as functional alterations such as downregulation of carbohydrate metabolic pathways (Figures 1, 2). These findings imply that viral infection may promote microbial reorganization by modifying intestinal microenvironmental conditions, particularly through changes in luminal pH and bile acid profiles. Notably, the persistent reduction in *B. fragilis* abundance exhibited a strong inverse correlation with viral load (Figure 2C–F). As a key commensal microorganism essential for maintaining intestinal barrier integrity and immune modulation, *B. fragilis* depletion may serve as both a biomarker and functional mediator of PDCoV pathogenesis [36, 37].

A key finding of our study was the significant alteration of bile acid profiles following PDCoV infection. Untargeted metabolomics revealed marked enrichment of primary and secondary bile acid synthesis pathways in infected piglets (Figure 3C). Subsequent targeted bile acid quantification demonstrated substantial reductions in unconjugated bile acids (CA, DCA, LCA) alongside elevated conjugated bile acids (GCA, GLCA) in PDCoV‐infected animals (Figure 3D,E). This metabolic shift likely results from impaired BSH activity due to *B. fragilis* depletion, as this commensal bacterium plays a pivotal role in bile acid deconjugation through its BSH enzyme. While *E. coli*'s negative association with PDCoV is intriguing, its exclusion from mechanistic studies reflects our focus on *B. fragilis*'s unique BSH‐dependent activity and its direct link to LCA‐mediated antiviral effects. Future studies could explore *E. coli*'s potential role in PDCoV pathogenesis through alternative pathways. Recent studies have found elevated levels of *B. fragilis* expressing BSH in the feces of patients with obesity‐associated colorectal cancer (CRC), where it activates the WNT/β‐catenin signaling pathway by increasing unconjugated bile acids, particularly DCA and LCA, thereby accelerating CRC progression [39]. Additionally, *B. fragilis* alleviates necrotizing enterocolitis by hydrolyzing bile salts via BSH activity, altering bile acid composition, regulating metabolic homeostasis, and affecting gut microbiota composition by inhibiting FXR activity [46]. The multifaceted role of *B. fragilis* highlights the importance of bile acid metabolism in disease progression and protection in various pathological contexts. The observed positive correlation between *B. fragilis* abundance and free bile acid levels (Figure 3I), coupled with its negative association with PDCoV viral load (Figure 2E,F), provides compelling support for this mechanistic link. Collectively, these findings demonstrate that PDCoV‐induced gut dysbiosis disrupts bile acid metabolism through suppression of microbial BSH activity. Colonization of BALB/c mice with BSH‐producing *B. fragilis* significantly increased unconjugated bile acids levels and reduced PDCoV infection, effects that were reversed by treatment with the BSH inhibitor GR‐7 (Figure 4C–F). These findings demonstrate that BSH activity plays a pivotal role in antiviral defense by modulating bile acid metabolism to suppress PDCoV replication. Wang et al. observed significant enrichment of LCA and DCA in the intestines of PEDV‐infected Min pigs, a premium pig breed from Northeast China, with LCA protecting against PEDV infection [20]. This phenomenon may be attributed to the robust immune defense system of Min pigs, which likely inhibits viral infection by upregulating antiviral bile acids. In our previous study, we noted a significant increase in bile acid concentrations (CA, CDCA, DCA, LCA, UDCA, TCDCA, and TLCA) in the small intestine following SADS‐CoV infection in piglets [18]. CA enhances SADS‐CoV entry in PIEs by inducing caveolae‐mediated endocytosis and endosomal acidification [18]. This bile acid‐rich environment in the small intestine rapidly establishes early SADS‐CoV infection and promotes its dissemination in small IECs. These findings highlight that specific structural and functional differences among coronaviruses may account for their varied responses to bile acids. While some coronaviruses may exploit bile acids to promote infection by altering cell membrane properties, others may be inhibited by bile acids that enhance host antiviral resistance.

A pivotal finding of this study is the potent antiviral effect of LCA against PDCoV. LCA effectively inhibited PDCoV infection in IPEC‐J2, PIEs (Figure 5), and in vivo (Figure 7). The broad‐spectrum antiviral activity of LCA highlights its potential as a therapeutic agent. Conventional understanding attributes bile acid‐mediated antiviral effects primarily to BAR signaling pathways. For example, prior studies have shown that bile acid treatment can upregulate SLA‐I expression via FXR to activate CD8 + T cell responses against PEDV infection [20]. However, our results reveal a distinct mechanism. While LCA significantly upregulated *FXR* and *TGR5* mRNA expression, pharmacological inhibition of FXR (using GUG) or TGR5 blockade (with SBI‐115) failed to reverse its antiviral effects (Figure S7). Crucially, LCA maintained robust antiviral activity even under dual receptor knockdown conditions, confirming its action through noncanonical pathways. This observation aligns with findings in SADS‐CoV studies, where bile acids enhanced viral replication via receptor‐independent mechanisms, indicating that certain bile acids may act beyond classical signaling paradigms [18]. Alternative pathways, such as those mediated by the pregnane X receptor, constitutive androstane receptor (CAR), sphingosine‐1‐phosphate receptor 2 (S1PR2), or vitamin D receptor, may contribute to LCA's antiviral effects. Supporting this hypothesis, a recent study demonstrated that bile acid‐dependent human norovirus GII.3 replication in human intestinal enteroids was independent of FXR or TGR5 signaling but involved S1PR2‐mediated viral particle uptake [9]. This receptor‐independent mechanism implies that LCA may remain effective even in scenarios where host immune responses are compromised or evaded by viral immune escape strategies.

Further mechanistic investigations revealed that LCA disrupts the viral entry process by inhibiting the interaction between PDCoV spike proteins and the cellular receptor APN (Figure 6). MDs simulations showed that LCA binding induces significant conformational changes in the RBD, potentially impairing its ability to interact with pAPN (Figure 6). These structural alterations likely underlie the observed inhibition of viral attachment. In contrast, bile acids enhance the binding affinity and infectivity of murine norovirus by stabilizing virus‐receptor interactions [47, 48, 49]. The differing effects of bile acids on PDCoV and murine norovirus can be attributed to the unique structural and functional characteristics of each virus and their respective interactions with host receptors or antiviral drugs. Understanding these differences is crucial for developing targeted antiviral therapies that exploit the vulnerabilities of each virus.

The zoonotic potential of PDCoV underscores the urgency of developing effective control and therapeutic strategies [33, 35]. Initially identified in swine, PDCoV exhibits broad tropism for APN molecules across diverse species, including chickens, mice, calves, and humans [22, 31, 35]. The binding modes of PDCoV to pAPN and hAPN are highly conserved, as the virus recognizes conserved residues on both receptors [50]. Notably, PDCoV demonstrates a higher binding affinity for hAPN than for pAPN [50], potentially facilitating more efficient zoonotic transmission. Genetic analyses have shown that PDCoV strains isolated from children in Haiti share significant homology with porcine isolates from China and the USA [33], highlighting the substantial risk of cross‐species transmission between pig and human. Importantly, our data demonstrate that LCA effectively inhibits PDCoV replication in hAPN‐expressing cells (Figure S11A,B). Molecular docking analyses identified specific hydrogen bond interactions between LCA and key residues (N397 in the viral RBD and N739 in hAPN) (Figure S11C–E), mirroring the binding pattern observed with pAPN. This conserved mechanism across species suggests that LCA's antiviral activity may be broadly applicable. By targeting the initial stages of viral infection, LCA could effectively mitigate the risk of interspecies transmission and reduce the likelihood of zoonotic spillovers of PDCoV, highlighting its potential as a broad prophylactic intervention for both humans and various animal species.

The successful translation of LCA's antiviral effects from in vitro models to in vivo models (Figure 7) is a critical step in developing new therapeutic interventions against PDCoV. Our experimental data demonstrate that oral administration of LCA (30 mg/kg/day) significantly reduced intestinal viral loads, alleviated pathological damage, and improved clinical symptoms in infected piglets (Figure 7). Notably, LCA treatment maintained intestinal homeostasis, as evidenced by normal expression patterns of gut barrier genes and absence of inflammatory or apoptotic signaling activation, indicating excellent enteric safety (Figure S12). These findings confirm LCA's molecular mechanism of targeting the interaction between PDCoV spike protein and pAPN while marking a crucial transition from in vitro inhibition to therapeutic application. Compared to other antiviral agents, such as IFN and protease inhibitors, LCA appears to offer a more targeted approach with fewer off‐target effects [51, 52, 53, 54]. LCA is a promising candidate for further development due to its specific action and potential to prevent viral entry. Future research will focus on optimizing dosing strategies, assessing long‐term safety, and conducting comparative efficacy studies.

Several limitations of this study should be noted. First, while we identified *B. fragilis* as a key contributor to BSH activity, our focus on this single species may not fully account for potential roles of other BSH‐producing gut microbes (e.g., *Sarcina perfringens*, *Lactobacillus spp*.) in bile acid metabolism. Second, the utilization of GR‐7, a broad‐spectrum BSH inhibitor, restricts our capacity to isolate *B. fragilis*‐specific BSH functions. Future studies employing *B. fragilis* BSH‐knockout strains or germ‐free mice could help dissect species‐specific contributions. Finally, although short‐term LCA administration exhibited no acute toxicity in piglets, its hydrophobicity and unresolved safety risks in chronic treatments demand further investigation. Prioritizing long‐term toxicity assessments, structural modifications, or targeted delivery systems may enhance its therapeutic potential.

## CONCLUSION

In summary, this study provides compelling evidence that PDCoV infection significantly alters the gut microbiota composition and metabolic profiles in piglets. We identified a crucial role of the commensal bacterium *B. fragilis* in modulating bile acid metabolism through BSH activity, leading to decreased unconjugated bile acid levels and concomitant accumulation of conjugated bile acids. LCA exhibited dose‐dependent antiviral effects in both IPEC‐J2 cells and intestinal organoid models. Mechanistically, LCA interferes with viral attachment by directly targeting the binding interface between the PDCoV spike protein and pAPN, independent of classical bile acid receptor signaling or host immune responses. Importantly, oral LCA administration in infected piglets significantly alleviated diarrhea, reduced intestinal viral loads, and maintained gut barrier integrity without inducing inflammatory responses, demonstrating its therapeutic potential and safety as an anti‐PDCoV candidate.

## METHODS

### Cell culture, virus, bacteria, and antibodies

HEK293T, IPEC‐J2, LLC‐PK1, and Vero cells were cultured in DMEM (C11995500BT, Gibco) supplemented with 10% (v/v) fetal bovine serum (FSD500, Excell), 100 U/mL penicillin, and 100 U/mL streptomycin. The cultures were maintained at 37°C in a 5% CO_2_ atmosphere with water‐saturated humidity. Vero cells stably expressing pAPN (Vero‐pAPN) were established in our laboratory using previously described methods [34]. To generate a pAPN‐deficient cell line (IPEC‐J2‐ΔpAPN), we employed a single‐plasmid CRISPR/Cas9 approach as described in previous studies [55]. The PDCoV strain used in this study was PDCoV HZYH‐2019 (GenBank accession: PQ645844). PDCoV viral titers were determined in LLC‐PK1 cells using endpoint dilution to calculate TCID_50_. *B. fragilis* was obtained from the American Type Culture Collection (ATCC, catalog no. 25285) and cultured in Chopped Meat Carbohydrate Broth at 37°C under anaerobic conditions. The anti‐PDCoV N monoclonal antibody (mAb) was purchased from Medgene Labs (Brookings, SD), while a polyclonal antibody (pAb) with broad reactivity to APN orthologues was developed in‐house [31]. The anti‐β‐actin (3700S, CST) mouse monoclonal antibody was purchased from Cell Signaling Technology.

### Animal experiments

Twelve 3‐day‐old piglets were randomly divided into two groups (*n* = 6). Fecal samples from these piglets were tested negative for PDCoV, PEDV, TGEV, and SADS‐CoV RNA by RT‐PCR as described previously [56]. Both groups were housed with their sows, which tested negative for PDCoV serum antibodies by ELISA and had not received artificial colostrum or milk supplementation. The PDCoV‐infected group was orally inoculated with 3 mL of PDCoV isolate (10⁶ TCID_50_/mL), whereas the Mock group received 3 mL of sterile DMEM using the same inoculation procedure. Piglets were monitored daily for clinical signs, including diarrhea and vomiting. At 3 dpi, all piglets were euthanized, and various tissues were collected for RNA extraction. Fecal samples were subjected to metabolomic profiling and 16S rRNA sequencing.

### Histopathology and IHC

Ileal tissue fixed in 4% paraformaldehyde was dehydrated, paraffin‐embedded, sectioned, mounted on slides, and stained with hematoxylin and eosin. Observations were conducted using an inverted fluorescence microscope. IHC was performed following established laboratory protocols [57]. PDCoV‐specific antigen detection was performed on selected paraffin‐embedded sections using a PDCoV‐N‐specific monoclonal antibody (1:500) and an HRP‐conjugated goat anti‐mouse IgG secondary antibody (1:1000).

### 16S rRNA gene sequencing and data analysis

Total microbial genomic DNA was extracted from fecal samples using the E.Z.N.A.® Stool DNA Kit (D4015, Omega Bio‐Tek) following the manufacturer's instructions. The extracted genomic DNA was detected using 1% agarose gel electrophoresis, and its concentration was measured on a NanoDrop 2000 spectrophotometer (Thermo Scientific). Variable regions 3 and 4 (V3–V4) of the 16S rRNA gene were amplified using TransStart Fastpfu DNA Polymerase (AP221‐02, TransGen) with fecal DNA as the template (338F: 5'‐ACTCCTACGGGAGGCAGCAG‐3'; and 806R: 5'‐GGACTACHVGGGTWTCTAAT‐3'). The PCR product was extracted from a 2% agarose gel and purified using the AxyPrep DNA Gel Extraction Kit (AP‐GX‐50) according to the manufacturer's instructions. The purified amplicons were pooled in equimolar amounts and subjected to paired‐end sequencing on an Illumina MiSeq platform (Illumina, SD) following standard protocols. Quality control of the paired‐end raw sequencing data was performed using FASTP (v0.24) [58]. Subsequently, the sequences were analyzed using the Quantitative Insights into Microbial Ecology (QIIME 2) platform (v2024.10). DADA2 was used to clean and merge the reads into exact amplicon sequence variants. Taxonomic assignment was conducted using the feature‐classifier plugin in QIIME2, referencing the SILVA (v138) database. Alpha‐diversity indices were computed via the *vegan* (v2.6‐10) and *picante* (v1.8.2) packages. To assess beta‐diversity, PCoA based on Bray‐Curtis distances was performed using the *vegan* package, and group differences were tested for significance using permutational multivariate analysis of variance (PERMANOVA, permutation = 999). The Wilcoxon rank‐sum test was used to measure significant differences in the relative abundance of microbial taxa between PDCoV‐infected and healthy piglets. LEfSe analysis was performed to identify microbial biomarkers using the *microeco* (v1.13) package. Spearman correlation analysis between the relative abundance of taxa and the viral loads was conducted utilizing *corr.test* function in the *psych* (v2.4.12) R package. For multiple testing, *p* values were adjusted by the BH procedure. All visualizations were produced via the *ggplot2* (v3.5.1) package in the R environment (v4.4.3).

### DNA extraction, library construction, and metagenomics sequencing

Genomic DNA was extracted from approximately 100 mg of each sample using the Magigene Stool DNA Kit following the manufacturer's instructions. Quality‐approved DNA samples were subjected to library preparation using the ALFA‐SEQ DNA Library Prep Kit following the manufacturer's protocols. The DNA was fragmented using sonication to achieve a size of approximately 350 bp, and then end‐polished, A‐tailed, and ligated with Illumina full‐length adapters, followed by PCR amplification. The libraries were pooled and sequenced on the BGISEQ‐400 platform with a PE150 strategy (Magigene Biotechnology Co., Ltd.).

### Metagenome bioinformatic analyses

The raw reads were filtered to remove unqualified reads using FASTP (v0.24) with options “‐q 20 ‐u 30 ‐l 80 ‐y”. The reads that aligned with the host genomic sequence (RefSeq accession: GCF_000003025.6) were removed using Bowtie2 (v2.5.4) with default parameters. The high‐quality reads were assembled for each sample by MEGAHIT (v1.2.9) [59]. Reads were mapped to contigs with a length >1500 bp using the minimap2 (v2.28‐r1209) [60]. We used the *single_easy_bin* pipeline in SemiBin2 (v2.1) with the options “–environment pig_gut” for binning. The quality of metagenomic bins was evaluated using CheckM2 (v1.1.0) [61, 62]. The MAGs were clustered using dRep (v3.5.0) with a threshold of average nucleotide identity (ANI) > 95%, resulting in 171 genome‐based species. Taxonomy assignment was performed using the standard workflow in GTDB‐Tk (v2.4.0) and the GTDB database (r220) [63]. PhyloPhlAn (v3.1.68) was used to construct a phylogenetic tree, which was visualized using iTOL (v7.1) [64]. Prokka (v1.14.6) was used to annotate genomic features for each MAG. The relative abundance of genome‐based species was profiled using minimap2 and CoverM (v0.7.0) with the default parameters. The relative abundance for higher taxonomic levels was determined by aggregating the abundances of their daughter clades. The protein‐coding gene sequences were clustered using the *easy‐cluster* workflow in MMseqs2 (v17‐b804f) with options “–cluster‐mode 2 –cov‐mode 1 –min‐seq‐id 0.95 ‐c 0.9 –kmer‐per‐seq‐scale 0.8” [65]. The representative protein‐coding genes were subjected to functional assignment by comparing them against the Kyoto Encyclopedia of Genes and Genomes (KEGG, release 106.0) database using DIAMOND (v2.1.11.165) with the parameters of “‐min‐score 60 ‐query‐cover 50” [66]. Gene abundance in each sample was profiled by mapping the high‐quality reads against the reference gene set using minimap2. Read counts in each sample were transformed to Transcripts Per Kilobase Per Million Mapped Reads (TPM). The abundances of KEGG Orthologous (KOs) were calculated based on the abundances of genes assigned to them. *MaAsLin2* (v1.20) R package was used to identify differentially abundant microbial species between groups [67]. Heatmaps were visualized via the *ComplexHeatmap* (v2.22) R package. Other statistical analyses were similar to those in 16S rRNA amplicon analyses [68].

### LC‐MS/MS

A 50 mg fecal sample was placed in a 2 mL centrifuge tube with a 6 mm diameter grinding bead. For metabolite extraction, 400 μL of extraction solution (methanol: water = 4:1, v/v) containing 0.02 mg/mL of the internal standard L‐2‐chlorophenylalanine was added. The samples were ground using a Wonbio‐96c frozen tissue grinder for 6 min at −10°C and 50 Hz, followed by low‐temperature ultrasonic extraction for 30 min at 5°C and 40 kHz. The samples were then incubated at −20°C for 30 min, centrifuged at 13,000 × *g* for 15 min at 4°C, and the supernatant was collected for LC‐MS/MS analysis. The samples were analyzed using ultrahigh performance liquid chromatography tandem Fourier transform mass spectrometry (UHPLC‐Q Exactive HF‐X, Thermo Fisher Scientific) on the LC‐MS/MS system. The UHPLC‐MS raw data were processed using Progenesis QI software for baseline filtering, peak identification, peak integration, retention time correction, and peak alignment. The resulting data matrix, containing sample names, *m*/*z* values, retention times, and peak intensities, was exported for further analysis. Metabolites were identified by searching databases, including HMDB (http://www.hmdb.ca/), Metlin (https://metlin.scripps.edu/), and the in‐house Majorbio Database (Majorbio Biotechnology Co., Ltd.). PLS‐DA was employed to identify differentially abundant metabolites between groups, utilizing the *opls* function with parameters “orthoI = 0” from the *ropls* R package (v1.38). Meanwhile, the Welch's *t*‐test was also used to assess significant differences in the log_10_‐transformed metabolite abundance between the two groups. A metabolite was considered significantly enriched if its VIP value exceeded 1 and its BH‐adjusted *p*‐value was <0.05. Furthermore, KEGG pathway enrichment analyses of differentially abundant metabolites were performed using the *enricher* function from the *ClusterProfiler* R package (v4.14.6) [69], utilizing the pathway‐metabolite relationships (http://rest.kegg.jp/link/pathway/compound).

### LC–MS/MS determination of serum bile acid concentrations

For LC‐MS/MS analysis, 50 μL of internal standard working solution (200 ng/mL) and 350 μL extraction solution (methanol) were added to serum samples. The mixture was vortexed for 30 s, sonicated at 5°C and 40 kHz for 30 min, stored at −20°C for 30 min, and centrifuged at 13,000 × *g* for 15 min at 4°C. After removing the supernatant, the pellet was dried under nitrogen. Then 100 μL of 50% acetonitrile was added, and the mixture was vortexed for 30 s, sonicated at 5°C and 40 kHz for 10 min, and centrifuged at 13,000 × *g* for 15 min at 4°C before collecting the supernatant for LC‐MS. The LC‐MS/MS analysis was conducted using an ExionLC AD system coupled with a QTRAP® 6500 + mass spectrometer (Sciex) at Majorbio Bio‐Pharm Technology Co., Ltd. The LC‐MS raw data were imported into Sciex OS software. All ion fragments were automatically identified and integrated using default parameters, and all integrations were manually verified. The metabolite concentrations in samples were calculated based on a linear regression standard curve. A one‐way ANOVA test was performed to assess significant differences in the log_10_‐transformed abundances of various bile acids between the two groups. Spearman correlation analysis between the relative abundances of microbial species and the bile acid concentrations was conducted utilizing *corr.test* function in the *psych* R package.

### BSH activity analysis

For the BSH activity assay with different conjugated bile acids, *B. fragilis* cultures were centrifuged at 12,000 × *g* for 2 min. Subsequently, the pellets were resuspended in 100 μL of 0.01 M phosphate buffer and then a 500 μM mixture of conjugated bile acids (TCA, TCDCA, TLCA). The mixture was incubated at 37°C for 60 min, and then the reactions were immediately terminated by adding 100 μL of 15% (w/v) trichloroacetic acid. Subsequently, the mixture was centrifuged at 12,000 × *g* for 10 min to obtain the reaction samples. The detection of deconjugated bile acids was performed using UPLC‐MS/MS.

In the ninhydrin chromogenic assay for measuring enzyme activity, the bacteria were resuspended in 0.01 M phosphate buffer, and a 500 μM mixture of conjugated bile acids (TCA, TCDCA, TLCA) was added. The mixture was incubated at 37°C for 60 min. To terminate the reaction, an equal volume of 15% trichloroacetic acid was added, and then the mixture was centrifuged at 4°C for 10 min at 12,000 × *g*. Subsequently, 100 μL of the supernatant was combined with 900 μL of ninhydrin chromogenic solution, boiled for 15 min, and the absorbance was measured at 570 nm. Standard curves were generated by diluting taurine (5 mM) to various concentrations. One unit of enzyme activity (U) was defined as the amount of enzyme required to produce 1 μM of amino acid per minute.

### Bacterial colonization and GR‐7 treatment

To establish colonization of *B. fragilis*, 6‐week‐old BALB/c mice were administered 200 μL of sterile anaerobic PBS containing 10^9^ CFU/mL of *B. fragilis* via gavage every 2 days. Following successful colonization, the mice were treated with GR‐7 at a dosage of 10 mg/kg via gavage for two consecutive days. Subsequently, each group of mice received an intraperitoneal injection of PDCoV (10^6^ TCID_50_/mL).

### Cell viability assay

IPEC‐J2 cells were seeded into 96‐well plates and incubated for 24 h with different concentrations of bile acids, agonists, and antagonists. Cell viability was subsequently evaluated using the CCK‐8 kit, and absorbance was recorded at 450 nm.

### Antiviral activity assay

The compounds were dissolved in dimethyl sulfoxide (DMSO) and diluted in cell culture medium before incubation with the cells. IPEC‐J2 cells were pretreated with varying concentrations of each compound at 37°C for 1 h. After pretreatment, the cells were infected with PDCoV at an MOI of 0.1 for 1 h. Following three washes with PBS, the cells were cultured in medium containing the compounds for an additional 24 h. Finally, IPEC‐J2 cells were harvested, and the viral loads were determined using RT‐qPCR.

### Investigation of bile acids on the growth kinetics of PDCoV

IPEC‐J2 cells were seeded into 24‐well plates and cultured until reaching 90% confluence. Cells were inoculated with PDCoV at an MOI of 0.1, premixed with LCA or DCA, and incubated for 2 h at 37°C. Following incubation, the unbound virus was removed by gentle washing with PBS. To assess sustained antiviral effects, the culture medium was replaced with fresh medium containing LCA/DCA, and cells were maintained in a humidified incubator (37°C, 5% CO₂). Supernatants and cells were collected at 2, 8, 12, 24, 48, and 72 hpi. The viral loads in cells were quantified using RT‐qPCR targeting the PDCoV M gene, and the infectious viral particle titer in supernatants was measured by TCID_50_ assays using LLC‐PK1 cells.

### Treatment with bile acid receptor agonists/antagonists

For the agonist experiment, a 50 μM concentration was applied to five treatment groups: vehicle control, LCA alone, and three agonist groups (INT‐777, INT‐747, INT‐767). In the antagonist experiment, safe inhibitor concentrations (25, 50, and 100 μM) were tested across three groups: vehicle control, antagonists alone, and antagonists co‐administered with 50 μM LCA. IPEC‐J2 cells seeded in 24‐well plates were cultured to 90% confluence. The original medium was replaced with serum‐free medium containing the designated drugs for 2 h. After pretreatment, cells were washed three times with ice‐cold PBS and inoculated with PDCoV (MOI = 0.1) in drug‐supplemented medium for 1 h at 37°C. Post‐adsorption, the viral inoculum was removed, and maintenance medium with corresponding drugs was added. Cells were incubated for 24 h postinfection, after which samples were harvested for analysis.

### RNA isolation and RT‐qPCR

Total RNA was extracted from cells or tissues using TRIzol (Thermo Fisher Scientific) according to the manufacturer's instructions. The RNA titers of PDCoV were determined by RT‐qPCR targeting the membrane (M) gene, using specific primers 5′‐ATCGACCACATGGCTCCAA‐3′ and 5′‐CAGCTCTTGCCCATGTAGCTT‐3′, and FAM‐labeled probe CACACCAGTCGTTAAGCATGGCAAGCT‐BHQ. The RT‐qPCR protocol was previously published [34, 70]. The mRNA expression levels of target genes were detected by SYBR Green RT‐qPCR, with *GAPDH* as the internal reference gene. Relative expression levels were calculated using the 2^−ΔΔCt^ method, and primer sequences are listed in Table S4.

### Immunofluorescence assay (IFA) and western blot analysis

Before the experiment, IPEC‐J2 or Vero‐pAPN cells were seeded in 24‐well plates at appropriate densities. The cells were infected with PDCoV at an MOI of 0.1 in the presence of different concentrations of LCA for 1 h at 37°C, followed by three washes with PBS. The cells were then incubated for an additional 24 h in fresh culture medium containing LCA to observe cellular responses and viral replication. For IFA, the cells were washed twice with PBS and fixed with 4% paraformaldehyde in PBS for 20 min, followed by permeabilization with 0.5% Triton X‐100 for 10 min. The primary antibody against PDCoV‐S (1:1000 dilution in PBS) was applied to the cells and incubated for 1 h at 37°C. After two washes with PBS, the cells were incubated with Alexa Fluor 488‐conjugated goat anti‐rabbit IgG (Thermo Fisher Scientific), followed by DAPI staining. For western blot analysis, the cells were lysed, and the lysate was mixed with protein loading buffer. The mixture was denatured by heating at 100°C for 5 min, then proteins were separated using SDS‐PAGE and transferred onto polyvinylidene difluoride membranes. The membranes were blocked overnight at 4°C with 5% nonfat milk to minimize nonspecific binding. After washing, the membranes were incubated overnight at 4°C with the primary antibody, followed by incubation with an HRP‐conjugated secondary antibody for 1 h at room temperature. Finally, protein bands were visualized using an enhanced chemiluminescence imaging system.

### PIE 3D and 2D culture

Porcine intestinal crypts were isolated from 2‐ to 7‐day‐old piglets following previously established protocols [18]. Briefly, freshly isolated porcine small intestinal crypts were mixed with an equal volume of Matrigel and DMEM/F12. The mixture was seeded at the center of preheated 24‐well plates in a vertical orientation. Plates were gently transferred to a 37°C incubator and incubated for 10 min to allow undisturbed solidification of the Matrigel domes. Subsequently, 500 μL of IntestiCult™ Organoid Growth Medium (#06010, STEMCELL) was carefully added to each well by pipetting along the well side to minimize disturbance.

To prepare mature organoids for experiments, the matrix gel was disrupted using a mild digestion solution and a 1‐mL pipette. Organoids were digested at room temperature for 10 min, followed by centrifugation at 200 × *g* for 5 min at 4°C to pellet the cells. The pellet was resuspended in 0.05% Trypsin‐EDTA and incubated for an additional 5–10 min at 37°C. Complete dissociation into single cells was ensured by vortexing. Digestion was stopped by adding DMEM/F12 containing 10% FBS, followed by a second centrifugation at 290 × *g* for 5 min at 4°C. The resulting cell suspension was carefully added to Matrigel‐coated 96‐well plates and resuspended in an intestinal monolayer growth medium. Cultures were incubated at 37°C with 5% CO₂. Daily monitoring confirmed growth, and organoids were typically ready for further experiments within 2–3 days.

### Inhibition assay at different infection stages

The impact of LCA on the PDCoV infection cycle was evaluated by infecting IPEC‐J2 cells at an MOI of 0.1 for 1 h at 37°C, followed by LCA treatment at three time points: 1 h before inoculation, during co‐incubation, or 1 h postinfection. To determine the specific stage at which LCA affects virus entry, LCA was added during the attachment or internalization stages of viral infection. For attachment assays, IPEC‐J2 cells were pre‐cooled to 4°C and incubated with a mixture of LCA and PDCoV at 4°C for 1 h. Cells were then washed three times with pre‐cooled PBS, supplemented with fresh medium, and incubated at 37°C for 24 h. For internalization assays, pre‐cooled cells were infected with PDCoV at 4°C for 1 h, washed three times with PBS, and incubated with LCA at 37°C for 1 h. After an additional wash with PBS, cells were cultured in medium at 37°C for 24 h before harvesting for RNA extraction and analysis.

### Pseudovirus inhibition assay

The pNL4‐3.Luc.RE backbone plasmid and PRK5‐PDCoV‐S plasmid were cotransfected into HEK293T cells. After 8 h of transfection, the medium was replaced with fresh medium containing 2% serum. At 48 h posttransfection, the supernatant was collected, centrifuged at 12,000 × *g* for 10 min at 4°C to remove cell debris, and stored at −80°C. The PDCoV pseudovirus was incubated with serial dilutions of LCA (6.5, 12.5, and 50 μM) for 30 min at 37°C. Vero‐pAPN cells were infected with the pretreated pseudovirus, incubated for 48 h at 37°C, washed with PBS, and lysed with lysis buffer. Luciferase activity was analyzed using a luminometer.

### Protein expression and purification

The PDCoV S protein trimer sequence with a His tag was amplified and cloned into the PRK5 eukaryotic expression vector. A mutant plasmid was generated by substituting R357 and N355 with alanine residues. The recombinant plasmid was transfected into 293F cells. After 72 h of transfection, the cell culture supernatant was collected and purified using nickel beads. The APN‐mFC fusion protein, expressed in 293T cells, was isolated from the cell culture supernatant using protein A‐sepharose beads. The protein purity and quality were assessed using SDS‐PAGE and western blot analysis, and protein concentration was determined using a BCA assay kit.

### Enzyme‐linked immunosorbent assay

ELISA was performed following established in‐house protocols [71]. The pAPN‐mFc fusion protein was diluted in the coating solution and added to the 96‐well plates at a concentration of 200 ng/well. The plate was incubated overnight at 4°C, then blocked with 5% BSA for 1 h to prevent nonspecific binding. The His‐tagged PDCoV spike protein was pre‐incubated with varying concentrations of LCA for 30 min at 37°C. DMSO and an anti‐APN antibody were used as negative and positive controls, respectively. After four washes with PBST, the mixtures were added to the wells. Following another four PBST washes, plates were incubated with a 1:5000 dilution of HRP‐conjugated anti‐His tag antibody (ab1187, Abcam) for 1 h. After washing, 3,3′,5,′‐tetramethylbenzidine substrate was added and incubated for 15 min at 37°C. The reaction was terminated with 2 M H_2_SO_4_, and the absorbance was measured at 450 nm using a microplate reader.

### Molecular docking

The three‐dimensional (3D) structure of the RBD‐pAPN protein complex (PDB ID: 7VPP) was obtained from the RCSB Protein Data Bank. AutoDock Tools version v1.5.6 was used to prepare the protein target by removing water molecules, adding hydrogen atoms, and converting the protein and ligand formats to pdbqt. The small molecule's SDF structure was retrieved from the PubChem database and converted to PDB format using OpenBabel. Molecular docking was performed using AutoDock Vina. Based on the RBD‐pAPN interaction interface, the grid box center was set at the centroid coordinates of the co‐crystallized ligand's binding domain. The Vina scoring function was used to calculate binding free energy, with the top 10 optimal conformations output for analysis. The best‐docked pose was visualized in PyMOL 2.4.1, with key amino acid residues annotated to illustrate intermolecular interactions.

### MD simulations

MD simulations were conducted using GROMACS 2020.3. The AMBER99sb‐ildn force field and General AMBER Force Field were employed for protein and ligand parametrization, respectively. The protein‐ligand complex was solvated in a cubic water box (TIP3P model) with a minimum distance of 1.0 nm between the protein and box boundaries. The system was neutralized by adding Na⁺/Cl^−^ ions, and the energy was minimized using the steepest descent algorithm (50,000 steps). Sequential equilibration was performed under NVT (100 ps, 300 K, V‐rescale thermostat) and NPT (100 ps, 1 bar, Parrinello‐Rahman barostat) ensembles to stabilize temperature and pressure. Production MD simulations (100 ns, 300 K, 1 bar) used a 2‐fs timestep with periodic boundary conditions. Nonbonded interactions were calculated via a 1.4 nm cutoff for van der Waals (Lennard‐Jones potential) and Particle Mesh Ewald (PME, 0.16 nm grid spacing) for electrostatics. Bond lengths were constrained by the LINCS algorithm. Trajectories were analyzed for stability (RMSD/Rg) using GROMACS integrated tools. The visual molecular dynamics software v1.9.3 and PyMOL v2.4.1 were used to display, analyze, and animate trajectories visually.

### Validation of metabolic effects in newborn piglets

Newborn piglets were randomly divided into four groups for a metabolite validation: Group 1 (blank treatment, uninfected), Group 2 (LCA treatment, uninfected), Group 3 (PDCoV‐infected control), and Group 4 (LCA treatment, infected with PDCoV). Before infection, Groups 2 and 4 received daily oral LCA (30 mg/kg) for 5 days, while Groups 1 and 3 received physiological saline. On Day 0, treatments were continued as assigned, and Groups 3 and 4 were orally inoculated with PDCoV, while Group 1 received oral DMEM. Daily body weight and clinical signs such as diarrhea and lethargy were monitored. Fecal samples were collected at specified intervals (e.g., Days 1, 3, and 5) for viral load analysis. At 5 dpi, all piglets were euthanized, and tissues, including duodenum, jejunum, ileum, and MLN, were collected for viral load analysis.

### Statistical analysis

The data are presented as the mean ± standard deviation unless otherwise stated. Normality was evaluated using the Shapiro‐Wilk test. Statistical analyses were performed using two‐tailed unpaired Student's *t*‐test and one (two)‐way ANOVA with Dunnett's or Tukey's multiple comparisons test for normally distributed data, and the Wilcoxon rank‐sum test for non‐normally distributed data. Statistical analysis for in vitro experiments was performed with GraphPad Prism 8 (GraphPad Software), with significance defined as *p* < 0.05. For in vivo experimental data, statistical analyses were performed using a linear mixed‐effects model implemented via the *lmer* function in the *lmerTest* R package (v3.1‐3). The model formula was specified as “value ~ group * DPI + (1 | animal)” and “value ~ group * region + (1 | animal).” Post hoc comparisons were conducted by computing estimated marginal means (EMMs) with the *emmeans* function in the *emmeans* R package (v1.11).

## AUTHOR CONTRIBUTIONS

**Ya‐Qing Zhang**: Writing—original draft; investigation; writing—review and editing; formal analysis; data curation; methodology; validation; visualization. **Bin Wang**: Investigation; writing—review and editing; software. **Yong‐Le Yang**: Investigation. **Jin‐Xin Meng**: Writing—original draft; writing—review and editing; methodology; data curation. **Meng‐Di Zhang**: Investigation. **Yi‐Ke Li**: Investigation. **Bo Dong**: Formal analysis. **Yanan Zhang**: Investigation. **Bo‐Wen Liu**: Investigation. **Dong Yang**: Investigation. **Chun‐Miao Ji**: Investigation. **Yao‐Wei Huang**: Conceptualization; funding acquisition; writing—original draft; writing—review and editing; resources; supervision. **Shu Jeffrey Zhu**: Conceptualization; supervision; investigation; funding acquisition; project administration; resources; writing—original draft; writing—review and editing.

## CONFLICT OF INTEREST STATEMENT

The authors declare no conflicts of interest.

## ETHICS STATEMENT

All animal experiments were strictly carried out and approved by the Institutional Animal Care and Use Committee (approval no. 25ZALAS17).

## Supporting information


**Figure S1.** Amplicon‐based analyses of the pig gut microbiota.
**Figure S2.** Shotgun metagenome‐wide analysis of pig gut microbiota.
**Figure S3.** Microbial bile acid metabolism gene expression and viral load‐bile acid correlations.
**Figure S4.** Cytotoxicity of bile acids and Takeda G protein‐coupled receptor 5 (TGR5)/farnesoid X receptor (FXR) modulators in porcine intestinal epithelial cell line (IPEC‐J2) cells.
**Figure S5.** Lithocholic acid (LCA) inhibits porcine deltacoronavirus (PDCoV) infection in porcine kidney cell line (LLC‐PK1) cells.
**Figure S6.** LCA‐mediated inhibition of PDCoV infection is independent of bile acid receptor signaling.
**Figure S7.** LCA‐mediated inhibition of PDCoV infection is independent of innate immune signaling.
**Figure S8.** LCA prevents PDCoV infection by disrupting the viral entry process.
**Figure S9.** Molecular docking results map.
**Figure S10.** Enzyme‐linked immunosorbent assay (ELISA) analysis of LCA‐mediated modulation of spike protein mutations and porcine aminopeptidase N (pAPN) interaction.
**Figure S11.** LCA inhibits the binding of PDCoV receptor‐binding domain (RBD) to human aminopeptidase N (hAPN).
**Figure S12.** Transcriptomic profiles of the ileal tissues from PDCoV‐infected piglets.
**Figure S13.** Expression of interferon (IFN) and IFN‐stimulated genes in PDCoV‐infected piglets.

**Table S1.** Microbial biomarkers identified by linear discriminant analysis (LDA) effect size (LEfSe) in 16S rRNA amplicon sequencing data.
**Table S2.** Summary of 479 metagenome‐assembled genomes (MAGs), including taxonomic classification, genomic features, and quality metrics.
**Table S3.** Differentially abundant microbial species identified by (Microbiome Multivariable Association with Linear Models) MaAsLin2 in shotgun metagenomic data.
**Table S4.** Primers used in this study.

## Data Availability

The data that support the findings of this study are openly available in the Genome Sequence Archive in the National Genomics Data Center at https://ngdc.cncb.ac.cn/, reference number CRA020669. The 16S rRNA amplicon and shotgun metagenome sequencing data reported in this paper have been deposited in the Genome Sequence Archive in the National Genomics Data Center under accession codes CRA020669 (https://ngdc.cncb.ac.cn/gsa/browse/CRA020669) and CRA024926 (https://ngdc.cncb.ac.cn/gsa/browse/CRA024926), respectively. Pig fecal and serum metabolome data reported have been deposited in the China National Center for Bioinformation under accession codes OMIX007990 (https://ngdc.cncb.ac.cn/omix/release/OMIX007990) and OMIX007989 (https://ngdc.cncb.ac.cn/omix/release/OMIX007989). The molecular docking and dynamics simulation files supporting this study have been deposited in the Figshare repository (https://doi.org/10.6084/m9.figshare.28794119.v1). The codes and supporting data related to this study are available on GitHub at https://github.com/mengjx855/25-PDCoV-BSH. Supplementary materials (figures, tables, graphical abstract, slides, videos, Chinese translated version, and update materials) may be found in the online DOI or iMeta Science http://www.imeta.science/.
