# Supplementary material for: A gut microbiota‐bile acid axis inhibits the infection of an emerging coronavirus by targeting its cellular receptor aminopeptidase N
Source: Imeta. 2025 Jul 3;4(5):e70061. doi: 10.1002/imt2.70061 (PMC12527990; doi:10.1002/imt2.70061)
Supplement: Supplementary file 1 — Figure S1. Amplicon‐based analyses of the pig gut microbiota. Figure S2. Shotgun metagenome‐wide analysis of pig gut microbiota. Figure S3. Microbial bile acid metabolism gene expression and viral load‐bile acid correlations. Figure S4. Cytotoxicity of bile acids and Takeda G protein‐coupled receptor 5 (TGR5)/farnesoid X receptor (FXR) modulators in porcine intestinal epithelial cell line (IPEC‐J2) cells. Figure S5. Lithocholic acid (LCA) inhibits porcine deltacoronavirus (PDCoV) infection in porcine kidney cell line (LLC‐PK1) cells. Figure S6. LCA‐mediated inhibition of PDCoV infection is independent of bile acid receptor signaling. Figure S7. LCA‐mediated inhibition of PDCoV infection is independent of innate immune signaling. Figure S8. LCA prevents PDCoV infection by disrupting the viral entry process. Figure S9. Molecular docking results map. Figure S10. Enzyme‐linked immunosorbent assay (ELISA) analysis of LCA‐mediated modulation of spike protein mutations and porcine aminopeptidase N (pAPN) interaction. Figure S11. LCA inhibits the binding of PDCoV receptor‐binding domain (RBD) to human aminopeptidase N (hAPN). Figure S12. Transcriptomic profiles of the ileal tissues from PDCoV‐infected piglets. Figure S13. Expression of interferon (IFN) and IFN‐stimulated genes in PDCoV‐infected piglets. [file IMT2-4-e70061-s001.docx]

**Supporting information to**

**A gut microbiota-bile acid axis inhibits the infection of a****n emerging coronavirus by targeting** **its cellular receptor aminopeptidase N**

**Running title:** Gut microbiota-bile acid axis hinders porcine deltacoronavirus infection

Ya-Qing Zhang^1,2#^, Bin Wang^1#^, Yong-Le Yang^3#^, Jin-Xin Meng^2#^, Meng-Di Zhang^3,4^, Yi-Ke Li^1,4^, Bo Dong^5^, Yanan Zhang^2^, Bo-Wen Liu^1^, Dong Yang^3^, Chun-Miao Ji^1^, Yao-Wei Huang^1,2,4*^, Shu Jeffrey Zhu^2*^

^1^State Key Laboratory for Animal Disease Control and Prevention, South China Agricultural University, Guangzhou, 510642, China

^2^Department of Veterinary Medicine, College of Animal Sciences, Zhejiang University, Hangzhou, 310058, China;

^3^Xianghu Laboratory, Hangzhou, 311231, China;

^4^Guangdong Laboratory for Lingnan Modern Agriculture, College of Veterinary Medicine, South China Agricultural University, Guangzhou, 510642, China;

^5^Agro-biological Gene Research Center of Guangdong Academy of Agricultural Sciences, State Key Laboratory of Swine and Poultry Breeding Industry, Guangzhou, 510640, China.

^#^ Ya-Qing Zhang, Bin Wang, Yong-Le Yang, and Jin-Xin Meng contributed equally to this study.

^*^Correspondence

[yhuang@zju.edu.cn](mailto:yhuang@zju.edu.cn) (Yao-Wei Huang), [shuzhu@zju.edu.cn](mailto:shuzhu@zju.edu.cn) (Shu Jeffrey Zhu)

**Supplementary figures**

**Figure S1.** **Amplicon-based metagenomic analyses of the pig gut microbiota. (A)** Diarrhea was observed in 5-day-old piglets on day 2 after inoculation with porcine deltacoronavirus (PDCoV) or Dulbecco’s Modified Eagle’s Medium (DMEM). **(B)** Gross pathology was assessed on day 5 following PDCoV or DMEM inoculation. **(C)** Boxplots of Faith’s phylogenetic diversity, Shannon diversity, and species richness indices for the pig gut microbiota in PDCoV-infected and control groups. Wilcoxon rank sum test: ^**^ *p* < 0.01. **(D)** PCoA scatter plot of gut microbiota β-diversity based on Bray-Curtis distances. PERMANOVA (1,000 permutations) tested the effect of PDCoV infection. **(E**−**F)** Relative abundance of pig gut microbiota at the **(E)** phylum and **(F)** genus levels in PDCoV-infected and control groups. **(G)** Linear discriminant analysis (LDA) effect size (LEfSe) analysis identifies microbial biomarkers distinguishing PDCoV-infected piglets from healthy controls. **(H)** Co-occurrence network of the bacterial genera correlated with viral loads across intestinal segments. Orange solid lines represent significant positive correlations, and green dashed lines represent significant negative correlations (*p* < 0.05). Circular nodes represent bacterial species (colored by phylum).

**Figure S2.** **Shotgun metagenome-wide analyses of pig gut microbiota. (A)** Boxplots of Faith’s phylogenetic diversity, Shannon diversity, and species richness indices for the pig gut microbiota in PDCoV-infected and control groups. Wilcoxon rank sum test: ^**^ *p* < 0.01. **(B)** Phylum-level composition of gut microbiota in PDCoV-infected and control groups. **(C)** Relative abundance of four dominant bacterial phyla (Pseudomonadota, Fusobacteriota, Bacillota_I, and Bacillota_A) in PDCoV-infected versus control piglets. Wilcoxon rank sum test: ^**^ *p* < 0.01; ^*^ *p* < 0.05. **(D)** Sunburst plot of Kyoto Encyclopedia of Genes and Genomes (KEGG) ortholog (KO) classification hierarchy, with sector sizes representing the number of KOs in each KEGG category. **(E)** Heatmap of metagenomic functional features across samples. Color intensity indicates z-score normalized abundance.

**Figure S3. Microbial bile acid metabolism gene expression and viral load-bile acid correlations.** The relative abundance (TPM) of bile salt hydrolase (*BSH*) (*K01442*) **(A)** and *baiCD* (*K15870*) genes **(B)** in the Mock group and PDCoV group. Wilcoxon rank sum test: ^**^ *p* < 0.01; n.s., not significant, *p* > 0.05. **(C)** Pearson correlation analysis between PDCoV viral loads and bile acid content.

**Figure S4. Cytotoxicity of bile acids and Takeda G protein-coupled receptor 5 (TGR5)/ farnesoid X receptor (FXR) modulators in porcine intestinal epithelial cell line (IPEC-J2) cells. (A)** Dose-dependent effects of bile acids on cell viability. **(B)** Viability of IPEC-J2 cells treated with the TGR5 antagonist (SBI-115) and FXR antagonist (guggulsterone, GUG). **(C)** Cell viability following treatment with TGR5/FXR agonists at the indicated concentrations.

**Figure S5**. **Lithocholic acid (LCA) inhibits porcine deltacoronavirus (PDCoV) infection in porcine kidney cell line (LLC-PK1) cells.** **(A, B)** Following PDCoV infection of LLC-PK1 cells, viral S protein was detected by immunofluorescence assay (IFA) (A), and viral N protein was detected by western blotting (B).

**Figure S6.** **LCA-mediated inhibition of PDCoV infection is independent of bile acid receptor signaling. (A**−**B)** mRNA levels of farnesoid X receptor (*FXR*) **(A)** and its downstream target small heterodimer partner (*SHP*) **(B)** in IPEC-J2 cells treated with LCA alone, the FXR agonist INT-747, the dual FXR/TGR5 agonist INT-767, the FXR antagonist GUG alone, or GUG combined with LCA. **(C)** mRNA levels of Takeda G protein-coupled receptor 5 (*TGR5*) and **(D)** intracellular cAMP levels in cells treated with LCA alone, TGR5 agonist INT-777, dual FXR/TGR5 agonist INT-767, TGR5 antagonist SBI-115 alone, or SBI-115 combined with LCA. One-way ANOVA with Tukey’s multiple comparisons test: ^****^ *p* ≤ 0.0001, ^***^ *p* < 0.001, ^**^ *p* < 0.01, ^*^ *p* < 0.05, n.s., not significant, *p* > 0.05. **(E-G)** Impact of FXR/TGR5 modulators on PDCoV infection: **(E)** PDCoV genome copies in IPEC-J2 cells treated with the FXR agonist INT-747, the TGR5 agonist INT-777, or the dual FXR/TGR5 agonist INT-767. One-way ANOVA with Tukey’s multiple comparisons test: ^***^ *p* < 0.001, n.s., not significant, *p* > 0.05. **(F, G)** PDCoV genome copies in cells treated with the FXR antagonist GUG **(F)** or the TGR5 antagonist SBI-115 **(G)**, with or without LCA. Two-way ANOVA with Tukey’s multiple comparisons test: ^****^ *p* ≤ 0.0001, not significant, *p* > 0.05. **(H**−**J)** siRNA-mediated knockdown of *FXR*/*TGR5*: Knockdown efficiency of *FXR* **(H)** and *TGR5* **(I)** mRNA after siRNA transfection. Two-tailed *t*-test: ^**^ *p* < 0.01, ^*^ *p* < 0.05. **(J)** PDCoV genome copies after FXR and TGR5 knockdown, with or without LCA. Two-way ANOVA with Tukey’s multiple comparisons test: ^****^ *p* ≤ 0.0001, not significant, *p* > 0.05.

**Figure S7.** **LCA-mediated inhibition of PDCoV infection is independent of innate immune signaling. (A**−**H)** Expression levels of innate immune markers in IPEC-J2 cells treated with: Mock (untreated control), Poly (I:C) (10 ng/mL, a synthetic dsRNA analog to activate innate immunity), LCA alone, PDCoV (MOI = 0.1) infection, or PDCoV + LCA (co-treatment). **(A**−**D)** mRNA levels of interferons (*IFNA*, *IFNB*, *IFNG*, and *IFNL1*) and **(E**−**H)** interferon-stimulated genes (*ISG15*, *IFITM1*, *MX1*, and *OAS1*) at 12 and 24 hpi. One-way ANOVA with Dunnett’s multiple comparisons test: ^****^ *p* ≤ 0.0001, ^***^ *p* < 0.001, ^**^ *p* < 0.01, ^*^ *p* < 0.05, n.s., not significant, *p* > 0.05. **(I**−**K)** Role of interferon receptors in LCA-mediated PDCoV suppression: **(I**−**J)** Knockdown efficiency of *IFNAR1* **(I)** and *IFNLR1* **(J)** mRNA after siRNA transfection. Two-tailed *t*-test: ^**^ *p* < 0.01. **(K)** PDCoV genome copies in *IFNAR1*/*IFNLR1*-depleted cells treated with LCA compared to scrambled siRNA controls. Two-way ANOVA with Tukey’s multiple comparisons test: ^****^ *p* ≤ 0.0001, not significant, *p* > 0.05.

**Figure S8.** **LCA prevents PDCoV infection by disrupting the viral entry process. (A)** Ileal organoids were infected with PDCoV (MOI = 0.1) and treated with LCA during three phases: pre-infection, co-treatment, or post-infection. Viral RNA loads were quantified by RT-qPCR at 24 hpi. **(B)** PDCoV-infected ileal organoids were incubated with LCA during attachment (4 °C) or internalization (37 °C) phases. Viral RNA loads were quantified by RT-qPCR at 24 hpi. Two-tailed *t*-test: ^****^ *p* ≤ 0.0001, n.s., not significant, *p* > 0.05. **(C)** Vero-pAPN cells were infected with PDCoV (MOI = 0.1) and treated with different concentrations of LCA for 24 h. Cells were then stained with a mouse anti-S protein antibody, followed by Alexa Fluor 488-conjugated anti-mouse IgG and DAPI for nuclei. Images were captured using a fluorescence microscope (scale bar: 100 μm). Relative fluorescence intensity was quantified using ImageJ software as shown on the right.

**Figure S9. Molecular docking results map. (A)** Docking diagram of DCA with the RBD-pAPN complexes. **(B)** Docking diagram of LCA with the R357A mutant complex.

**Figure S10.** **Enzyme-linked immunosorbent assay (ELISA) analysis of LCA-mediated modulation of Spike protein mutations and porcine aminopeptidase N (pAPN) interaction. (A, B)** The impact of LCA on the interaction between spike protein mutants R357A **(A)** and N355A **(B)** sites and the pAPN protein was assessed using the ELISA method. One-way ANOVA with Dunnett’s multiple comparisons test: ^****^ *p* ≤ 0.0001, ^***^ *p* < 0.001, n.s., not significant, *p* > 0.05.

**Figure S11.** **LCA inhibits the binding of PDCoV receptor-binding domain (RBD) to human aminopeptidase N (hAPN). (A)** PDCoV viral load in BHK-21 cells overexpressing hAPN and treated with LCA (10 μM). Two-tailed *t*-test: ^****^ *p* < 0.0001. **(B)** PDCoV infection was detected by IFA in BHK-21 cells overexpressing hAPN and treated with LCA. **(C**−**E)** Molecular docking of LCA with the PDCoV RBD-hAPN complex. **(C)** Global interaction diagram. **(D)** Local interaction diagram showing LCA (green) forming hydrogen bonds with N397 (RBD) and N739 (hAPN). **(E)** 2D interaction map highlighting key residues.

**Figure S12. Transcriptomic profiles of the ileal tissues of PDCoV-infected piglets. (A**−**C)** Expression of (A) intestinal barrier-related genes, **(B)** inflammation-related genes, and **(C)** apoptosis-related genes in the ileum. Significance was assessed by an unpaired two-tailed *t*-test: n.s., not significant, *p* > 0.05.

**Figure S13. Expression of interferon (IFN) and IFN-stimulated genes following PDCoV infection in piglets. (A**−**H)** The relative expression levels of *IFN* and its stimulated genes (*ISG15*, *IFITM1*, *MX1*, and *OAS1*) in the ileal tissues of piglets at 5 dpi. One-way ANOVA with Tukey’s multiple comparisons test: ^****^ *p* ≤ 0.0001, ^***^ *p* < 0.001, ^**^ *p* < 0.01, ^*^ *p* < 0.05, n.s., not significant, *p* > 0.05.
